# Genome-Wide Association Studies with a Genomic Relationship Matrix: A Case Study with Wheat and *Arabidopsis*

**DOI:** 10.1534/g3.116.034256

**Published:** 2016-08-11

**Authors:** Daniel Gianola, Maria I. Fariello, Hugo Naya, Chris-Carolin Schön

**Affiliations:** * Department of Animal Sciences, University of Wisconsin-Madison, Wisconsin 53706; † Department of Dairy Science, University of Wisconsin-Madison, Wisconsin 53706; ‡ Department of Biostatistics and Medical Informatics, University of Wisconsin-Madison, Wisconsin 53706; § Technical University of Munich School of Life Sciences Weihenstephan, Technical University of Munich, D-85354 Freising, Germany; ** Institute for Advanced Study, Technical University of Munich, D-85748 Garching, Germany; †† Bioinformatics Unit, Institut Pasteur de Montevideo, 11400, Uruguay; ‡‡ Instituto de Matemática y Estadística Rafael Laguardia, Facultad de Ingeniería, Universidad de la República, 11300 Montevideo, Uruguay

**Keywords:** GWAS, genomic relationship, heritability, whole-genome regression

## Abstract

Standard genome-wide association studies (GWAS) scan for relationships between each of *p* molecular markers and a continuously distributed target trait. Typically, a marker-based matrix of genomic similarities among individuals (**G**) is constructed, to account more properly for the covariance structure in the linear regression model used. We show that the generalized least-squares estimator of the regression of phenotype on one or on *m* markers is invariant with respect to whether or not the marker(s) tested is(are) used for building **G,** provided variance components are unaffected by exclusion of such marker(s) from **G**. The result is arrived at by using a matrix expression such that one can find many inverses of genomic relationship, or of phenotypic covariance matrices, stemming from removing markers tested as fixed, but carrying out a single inversion. When eigenvectors of the genomic relationship matrix are used as regressors with fixed regression coefficients, *e.g.*, to account for population stratification, their removal from **G** does matter. Removal of eigenvectors from **G** can have a noticeable effect on estimates of genomic and residual variances, so caution is needed. Concepts were illustrated using genomic data on 599 wheat inbred lines, with grain yield as target trait, and on close to 200 *Arabidopsis thaliana* accessions.

The advent of an enormous amount of DNA markers has given impetus to thousands of genome-wide association studies (GWAS) in humans, plants, and livestock (Yu *et al.* 2006; Manolio *et al.* 2009; Brachi *et al.* 2011; Gondro *et al.* 2013; Lipka *et al.* 2015); Neimann-Sorensen and Robertson (1961) represents one of the earliest searches for association between markers (blood groups in their study) and quantitative traits in animal genetics.

The most prevalent statistical method used in GWAS has been ordinary least-squares (OLS) linear regression of some phenotypic measurement on the number of copies of a reference allele at a single marker locus, *i.e.*, single marker regression (SMR). This method was subsequently enhanced by use of mixed linear model methodology, originally developed in animal breeding by Henderson (1948), with the purpose of accounting for correlated observations due to genetic or genomic similarities among individuals. Ignoring such correlations, as is done in OLS or in standard logistic regression, overstates precision and creates bias in populations undergoing artificial selection (Henderson 1975). These expectations from mixed model theory were corroborated prior to the GWAS wave by Kennedy *et al.* (1992) using a model where individuals were genotyped for a known gene; the genetic resemblance among individuals in the sample was accommodated using a random factor (additive effects) with levels that were correlated according to the infinitesimal model. In the SMR-GWAS context, Aulchenko *et al.* (2007) and Meyer and Tier (2012) also used an additive infinitesimal random effect with covariance matrix proportional to a pedigree-based kinship matrix, **A** (Henderson 1976). Given that molecular markers have become increasingly available, it was then natural to consider replacing **A** by a genome-based matrix **G** constructed using pairwise similarities in state between individuals. Variants of this type of matrix—called genomic relationship matrices—are used widely for SNP-based analysis in animal or plant breeding and human genetics (Nejati-Javaremi *et al.* 1997; Yu *et al.* 2006; Van Raden 2008; Astle and Balding 2009; Yang *et al.* 2010; Price *et al.* 2010; Legarra 2015). Along these lines, Teyssèdre *et al.* (2012) used analysis and simulation to study Type I error and power behavior of four models for GWAS; their conclusion was that the performance of SMR-OLS degraded relative to generalized least-squares as heritability and variability of relationships among individuals increased.

The preceding methods were also adopted and enhanced by human geneticists. Price *et al.* (2010) pointed out that how best to construct **G** in order to perform GWAS in some optimal manner, *e.g.*, to account for population stratification, was unclear. Consider the following question: if marker *j* in a GWAS is tested as a fixed effect in a SMR mixed model that includes **G** in the covariance structure, should the contribution of such marker to genomic similarity be removed from **G**? It is well understood (*e.g.*, Goddard 2009) that if a marker effect is treated as random then it contributes to the covariance structure, but it is not a location parameter of the phenotypic distribution, since the mean vector of the latter does not depend on zero-mean random effects. Hence, if a SMR model using **G** treats a marker as fixed and tests for its effect, the test would not appear to be net, as the marker is also contributing to variance, *i.e.*, it is implicitly included in the model as a random effect. In other words, the marker is viewed as having a fixed and a random effect simultaneously. The contradiction is clear: if a marker effect is a fixed parameter, it cannot have a frequentist variance. Consider a GWAS scan involving *p* markers; if the marker to be tested were to be removed when building up **G**, *p* distinct genomic relationship or phenotypic covariance matrices (of size *n* × *n* each) would need to be constructed and inverted (depending on the algorithm). The analysis would be impractical if the number of variants tested is large, as is the case with sequence data. It is fairly obvious that if *p* is very large, the impact of removing a marker should be nil. However, the question posed above deserves an unambiguous answer.

A similar issue arises in many treatments of genome-derived population structure presented in the literature. In principle, substructure must be accounted for in a GWAS somehow so that the analysis informs about association in a conceptually homogeneous population (Yu *et al.* 2006; Zhu and Yu 2009). Yang *et al.* (2010, 2011) accounted for population structure by extracting principal components (PC) from **G**, and regressions on a subset of these were regarded as fixed in a mixed model, but with **G** used without any modification; Stahl *et al.* (2012) presents an application. Janss *et al.* (2012) argued that such an approach would be ill-posed because it produces double counting: the eigenvectors of **G** used as covariates in the fixed part of the model are also an implicit part of **G**. Should **G** then be left intact? On one hand, the view could be taken that if an eigenvector is used as a covariate with a fixed regression coefficient, its contribution to **G** should be discounted. On the other hand, removal of the eigenvector could degrade the measure of similarity among individuals. Janss *et al.* (2012) pointed out that the eigen-decomposition of **G** would provide a solution to the problem (attenuation via eigenvalues) when the aim is to infer marker effects and genomic heritability. However, such attenuation shrinks all regressions on eigenvectors to 0 (to distinct degrees), and shrinkage on regressions on markers with medium or large effect sizes, or on eigenvectors used to account for population stratification, should not be exerted. Under such reasoning, the contribution to **G** of a marker or of eigenvector(s) associated with fixed regressions, should be discounted if one seeks net effect size estimates and corresponding tests of hypotheses. Another view (Astle and Balding 2009; Rincent *et al.* 2014) is that **G** conveys information on both population structure and relatedness, so it may “not be useful to consider admixture information as fixed effects covariates.” The preceding discussion reflects a lack of consensus in the GWAS field.

In this paper, we address the construction of **G** in a GWAS context. First, we describe how a single marker GWAS with, say, *p* conveniently constructed **G** matrices, can be carried out using a mixed linear model in which the marker effect tested is treated as fixed and the remaining *p* − 1 markers are used to introduce similarities in state, *i.e.*, *p* − 1 markers are viewed as having zero-mean random effects. A similar approach is discussed for the situation where eigenvectors are chosen as regressors (with fixed regression coefficients), with the purpose of accounting for population stratification, with the remaining eigenvectors used to induce similarities among individuals. In particular, we show algebraically that the generalized least-squares estimator of the regression on a marker is invariant with respect to whether or not the marker (or set of markers) treated as fixed is used when building **G**. It is also shown that removal of eigenvectors does matter. The manuscript is organized as follows. Basic concepts of GWAS conducted with kinship matrices in the model are reviewed in the sections under *Standard Approaches Used in GWAS*. Next, in *Impact of Removing Markers from the G-Matrix*, it is shown how inverses of the *p* needed genomic relationship (or of phenotypic covariance) matrices can be found in a convenient manner, and use the results to prove the invariance indicated above; removal of eigenvectors is also discussed in this section. To illustrate main, as well as related, concepts, data on wheat inbred lines and on *A. thaliana* were used, as described in *Case Studies with Wheat and Arabidopsis Data*. Technical details are shown in *Appendices* to the paper and toy examples are in Supplemental Material, File S1.

## Standard Approaches Used in GWAS

### Ordinary least-squares SMR

Let the *n* × *p* marker matrix be **X** = {*x_ij_*}; its *j^th^* column **x***_j_* (*j* = 1,2,…,*p*) contains marker genotype codes, *i* denotes individual, and *n* is sample size. Marker (SNP) genotypes can be coded as 0,1,2 for *aa*, *Aa*, *AA* individuals, respectively, where *A* is a reference allele; such coding captures additive genetic effects in the main. Markers and phenotypes are typically centered (*e.g.*, deviated from the mean), and most GWAS studies use the SMR model

$$y_{i}=x_{ij}\beta_{j}+e_{i};\;i=1,2,\ldots ,n;\;j=1,2,\ldots ,p,$$ (1)

where $y_{i}$ is the phenotype of individual i; $x_{ij}$ is the centered number of copies of the reference allele at locus *j* carried by i; $\beta_{j}$ is the fixed linear regression of $y_{i}$ on number of alleles at locus j, and $e_{i}∼NIID\left(0,\sigma_{e}^{2}\right)$ is a residual with variance $\sigma_{e}^{2}$; an intercept and additional nuisance effects (*e.g.*, smoking, age, and region) can be included in the model but these are not needed for the purposes of this discussion. NIID denotes that the SMR model assumes that residuals are normal, independent (an incorrect assumption if individuals are molecularly or genetically similar, or aggregated in families or spatially), and identically distributed. Notably, the SMR model postulates that the only effect affecting the mean of the distribution of *y*, given marker genotypes, is that of the SNP in question, hopefully flagging some genomic region in an unambiguous manner (the assumption is unlikely to hold for a complex trait).

The OLS estimator of the regression of *y* on the number of copies of allele *A* is

$$\beta_{j}^{SMR}=\frac{{x^{\prime }}_{j}y}{{x^{\prime }}_{j}x_{j}},$$ (2)

where ${x^{\prime }}_{j}x_{j}=\sum_{i=1}^{n}x_{ij}^{2}$ and ${x^{\prime }}_{j}y=\sum_{i=1}^{n}x_{ij}y_{i}$. Its variance is $Var\left(\beta_{j}^{SMR}\right)={\left({x^{\prime }}_{j}x_{j}\right)}^{-1}\sigma_{e}^{2}.$ With $\sigma_{e}^{2}$ estimated in some manner as ${\hat{\sigma }}_{e}^{2}$, the p−value for assessing significance of the regression is based on the statistic

$$t_{j}^{SMR}=\frac{\beta_{j}^{SMR}}{\sqrt{{\left({x^{\prime }}_{j}x_{j}\right)}^{-1}{\hat{\sigma }}_{e}^{2}}}=\beta_{j}^{SMR}\sqrt{\frac{\sum_{i=1}^{n}x_{ij}^{2}}{{\hat{\sigma }}_{e}^{2}}}.$$ (3)

The OLS-based test is anticonservative: the standard error is understated if important location and dispersion effects are ignored (Henderson 1984; Kennedy *et al.* 1992; Teyssèdre *et al.* 2012). Hence, *p* values must be taken with caution when a complex trait is confronted because of model specification error. Further, family effects or genomic similarities among individuals affect the variance–covariance structure of the observations, and OLS SMR ignores this issue. Because of the assumption of independence of residuals, the SMR approach sees more statistical information in the data set than there actually is.

### Generalized least-squares (GLS) SMR using a matrix of realized genomic relationships

Write the raw marker genotypes as $X=\left[x_{1},x_{2},\ldots ,x_{p}\right],$ with $x_{j}$ as before. A genomic similarity or relationship matrix G of order n×n can be formed as

$$G=\text{XX′}=\sum_{j=1}^{p}x_{j}{x^{\prime }}_{j};$$ (4)

Observe that G is the sum of *p* matrices of order n×n, each representing the contribution of a given marker to relatedness. Assume, without loss of generality, that **G** is positive definite, and that it has rank *n*. If p<n, **G** does not possess a unique inverse as its rank would be *p* at most. If *p* is large, the contribution of marker *j* to diagonal and off-diagonal elements of **G** is negligible relative to that made by the other p−1 markers.

An improvement over OLS-SMR uses realized relationships in the regression model, to account for correlations between individuals. With markers, one can observe variable degrees of similarity between full-sibs, that differ from, say, the expected additive relationship of 1/2, depending on the actual alleles inherited (Hill and Weir 2011). This feature of **G** renders the GWAS model more effective because similarities among individuals are represented in a more informed manner. Here, the regression model (1) is augmented with a random genomic effect $g_{i}$ as follows:

$$y_{i}=x_{ij}\beta_{j}+g_{i}+e_{i},$$ (5)

where $g_{i}∼N\left(0,\sigma_{g}^{2}\right)$ is the part of the additive genetic effect of individual *i* (assumed to vary at random in the population) that is captured by all *p* markers; $\sigma_{g}^{2}$ is a genomic variance component. If $e_{i}$ and $g_{i}$ are independent, the narrow sense genomic heritability is $h_{g}^{2}=\frac{\sigma_{g}^{2}}{\sigma_{g}^{2}+\sigma_{e}^{2}},$ where $\sigma_{y}^{2}=\sigma_{g}^{2}+\sigma_{e}^{2}$ is the phenotypic variance (Yang *et al.* 2010; de los Campos *et al.* 2015). In vector form, put $g∼N\left(0,G\sigma_{g}^{2}\right)$.

Let $V=G\sigma_{g}^{2}+I\sigma_{e}^{2}$. Under (5) the GLS estimator of $\beta_{j}$ is

$$\beta_{j}^{G}=\frac{{x^{\prime }}_{j}V^{-1}y}{{x^{\prime }}_{j}V^{-1}x_{j}},$$ (6)

with

$$Var\left(\beta_{j}^{G}\right)={\left[{x^{\prime }}_{j}V^{-1}x_{j}\right]}^{-1}=\sigma_{e}^{2}{\left[{x^{\prime }}_{j}{\left(V^{G}\right)}^{-1}x_{j}\right]}^{-1},$$ (7)

where *G* means that genomic relationships enter into the phenotypic variance–covariance structure and $V^{G}=G\frac{h_{g}^{2}}{1-h_{g}^{2}}+I$. No obvious computational advantage results from using the mixed model equations for the purpose of obtaining either the GLS estimator of $\beta_{j}$ or BLUP(g), where $g=\left\{g_{i}\right\}$ is the vector of marked additive genetic values after accounting for the regression of $y_{i}$ on $x_{j}$; BLUP means best linear unbiased predictor (knowledge of $h_{g}^{2}$ is needed). A standard (Searle 1974) representation of genomic BLUP gives

$$BLUP\left(g\right)=\sigma_{g}^{2}GV^{-1}\left(y-x_{j}\beta_{j}^{G}\right)=\frac{h_{g}^{2}}{1-h_{g}^{2}}G{\left(V^{G}\right)}^{-1}\left(y-x_{j}\beta_{j}^{G}\right).$$ (8)

Whether or not G has a unique inverse is immaterial because $V^{G}$ is invertible: BLUP(g) is unique irrespective of rank deficiency in G, and the GLS estimator $\beta_{j}^{G}$ is unique as well.

## Impact of Removing Markers from the G-Matrix

### General considerations

As pointed out earlier, if a regression on a marker is treated as a fixed effect, it would seem sensible to remove its contribution to G. Otherwise, there would be a contradiction: a fixed effect affects the mean of a distribution but does not contribute to covariance structure. Conversely, a zero-mean random effect contributes to dispersion (variance and covariance) but not to location (*e.g.*, Henderson 1984; Gianola 2013)

Conceivably, a marker effect could be modeled as the sum of a fixed and of a random component; the fixed part would index the mean of the distribution, and the random part would contribute to the likelihood only through covariance structure. This view, however, contradicts quantitative genetic theory, where quantitative trait locus (QTL) effects are fixed and genotypes are random (*e.g.*, Falconer and Mackay 1996; Lynch and Walsh 1998; Gianola *et al.* 2009; Gianola 2013; de los Campos *et al.* 2015). In classical quantitative genetics, QTL effects do not have variance but these loci generate variance in allelic content among individuals. On the other hand, Bayesian regression models pose a variance on effects that reflects uncertainty, *a priori*. Actually, the Bayesian treatment of a fixed effect (*e.g.*, a flat prior) implies an infinite prior variance provided the flat prior is unbounded. Here, we examine the question of whether or not a marker effect declared as fixed can also be allowed to have a random effect, as implied when including the marker in question in the building of **G**.

### GWAS using the GLS representation

If the effect of marker *j* is fixed in the GWAS, and the marker is removed when constructing **G**, *p* distinct genomic relationship matrices need to be built to carry out the GLS GWAS, accordingly, with *p* V or $V^{G}$ matrices formed and inverted. This procedure is computationally taxing if *p* is large. A short-cut is described below, with the result used subsequently to show that the GWAS can actually be carried out using V without modification.

Let $G_{\left[-j\right]}$ be an n×n genomic relationship matrix constructed without using marker j, built with the remaining p−1 markers. The SMR-GLS model is

$$y_{i}=x_{ij}\beta_{j}+g_{i,-j}+e_{i},$$ (9)

where $g_{i,-j}$ is the marked additive genomic value of *i* using the p−1 markers other than *j* in $G_{\left[-j\right]}.$ In an obvious vector notation, the model becomes

$$y=x_{j}\beta_{j}+g_{\left[-j\right]}+e=x_{j}\beta_{j}+\varepsilon_{\left[-j\right]},$$ (10)

where $\varepsilon_{\left[-j\right]}=g_{\left[-j\right]}+e$. Under independence of $g_{\left[-j\right]}$ and e

$$Var\left(\varepsilon_{\left[-j\right]}\right)=G_{\left[-j\right]}\sigma_{g}^{2}+I\sigma_{e}^{2}=V_{\left[-j\right]};j=1,2,\ldots ,p,$$ (11)

and $\sigma_{g}^{2}$ is the marked additive genetic variance. For simplicity, assume that exclusion of marker *j* from **G** does not change $\sigma_{g}^{2}$ and $\sigma_{e}^{2}$ appreciably; this is reasonable if *p* is large, the marker minor allele is rare and the substitution effect is small. Minor perturbations in values of variance components have little impact on GLS estimates of fixed effects because the latter depends on variance ratios only, at least in single trait models (Henderson 1984). The GLS estimator is now

$${\hat{\beta }}_{j}=\frac{{x^{\prime }}_{j}V_{\left[-j\right]}^{-1}y}{{x^{\prime }}_{j}V_{\left[-j\right]}^{-1}x_{j}},$$ (12)

with variance

$$Var\left({\hat{\beta }}_{j}\right)={\left({x^{\prime }}_{j}V_{\left[-j\right]}^{-1}x_{j}\right)}^{-1}.$$ (13)

This representation requires inverting each of the n×n $V_{\left[-j\right]}^{-1}$ matrices for implementing the procedure, which is unfeasible for dense marker platforms (*e.g.*, hundreds of thousands or millions of markers), even if *n* is moderate. However, use of (47) in *Appendix A* produces

$$V_{\left[-j\right]}^{-1}={\left(V-x_{j}{x^{\prime }}_{j}\sigma_{g}^{2}\right)}^{-1}=V^{-1}+\frac{\sigma_{g}^{2}V^{-1}x_{j}{x^{\prime }}_{j}V^{-1}}{1-\sigma_{g}^{2}{x^{\prime }}_{j}V^{-1}x_{j}};\;\;j=1,2,\ldots ,p.$$ (14)

Putting $t_{j}=V^{-1}x_{j}$

$$V_{\left[-j\right]}^{-1}=V^{-1}+\frac{\sigma_{g}^{2}t_{j}{t^{\prime }}_{j}}{1-\sigma_{g}^{2}{x^{\prime }}_{j}t_{j}};\;\;j=1,2,\ldots ,p.$$ (15)

Thus, the problem of computing *p* inverses is replaced by one involving a single inversion plus a series of matrix multiplications. Toy examples are in File S1.

Does it make a difference whether or not marker *j* is used or excluded when building **G**? Consider two GLS estimators: one with and the other without marker *j* included in **G**. Let these estimators be ${\hat{\beta }}_{j,\text{in}}$ and ${\hat{\beta }}_{j,\text{out}}$, respectively; the corresponding inverses of the phenotypic variance-covariance matrices are $V^{-1}$ and $V_{\left[-j\right]}^{-1}$. Assume that variance components are not affected appreciably by exclusion of the marker from **G**. The difference between the two GLS estimators is

$$\Delta_{\beta_{j}}={\hat{\beta }}_{j,\text{in}}-{\hat{\beta }}_{j,\text{out}}=\frac{{x^{\prime }}_{j}V^{-1}y}{{x^{\prime }}_{j}V^{-1}x_{j}}-\frac{{x^{\prime }}_{j}V_{\left[-j\right]}^{-1}y}{{x^{\prime }}_{j}V_{\left[-j\right]}^{-1}x_{j}}.$$ (16)

We show in *Appendix B* that $\Delta_{\beta_{j}}=0,$ *i.e.*, exclusion of marker *j* when forming **G** does not affect the generalized least-squares estimator. This result holds provided that **G** is built consistently with the way in which $x_{j}$ has been coded, *i.e.*, the $x_{j}$ in $G=\sum_{j=1}^{p}x_{j}{x^{\prime }}_{j}$ must be the same as the $x_{j}$ used as covariate. The surprising result that $\Delta_{\beta_{j}}=0$ has not been reported hereto.

Even though the GLS estimator can be computed in the usual form, a subtle point is that ${\left({x^{\prime }}_{j}V^{-1}x_{j}\right)}^{-1}=s_{j}^{-1}$ does not give the correct variance under the assumption that the effect of marker *j* is treated as fixed. The variance is

$$Var\left({\hat{\beta }}_{j,\text{out}}\right)=Var\left(\frac{{x^{\prime }}_{j}V^{-1}y}{{x^{\prime }}_{j}V^{-1}x_{j}}\right)=\frac{1}{s_{j}^{2}}{x^{\prime }}_{j}V^{-1}V_{\left[-j\right]}V^{-1}x_{j}.$$ (17)

### GWAS and BLUP using the mixed model representation

For model (10), an alternative way of computing ${\hat{\beta }}_{j}$ is via the mixed model equations, given the variance ratio $\lambda_{g}=\frac{\sigma_{e}^{2}}{\sigma_{g}^{2}}$. The linear system of equations to be solved is

$$\left[\begin{array}{cc} {x^{\prime }}_{j}x_{j} & {x^{\prime }}_{j}\\ x_{j} & I+G_{\left[-j\right]}^{-1}\lambda_{g} \end{array}\right]\left[\begin{array}{c} {\hat{\beta }}_{j}\\ {\hat{g}}_{\left[-j\right]} \end{array}\right]=\left[\begin{array}{c} {x^{\prime }}_{j}y\\ y \end{array}\right];\;\;j=1,2,\ldots ,p.$$ (18)

Above ${\hat{\beta }}_{j}$ is the GLS estimator, and ${\hat{g}}_{\left[-j\right]}$ is the BLUP of $g_{\left[-j\right]}$; $G_{\left[-j\right]}^{-1}$, with order n×n, would need to be computed for each single marker regression scan. Further, letting

$${\left[\begin{array}{cc} {x^{\prime }}_{j}x_{j} & {x^{\prime }}_{j}\\ x_{j} & I+G_{\left[-j\right]}^{-1}\lambda_{g} \end{array}\right]}^{-1}=\left[\begin{array}{cc} c^{\beta \beta } & {\left(c^{\beta g_{\left[-j\right]}}\right)}^{\prime }\\ c^{\beta g_{\left[-j\right]}} & C^{g_{\left[-j\right]}g_{\left[-j\right]}} \end{array}\right],$$ (19)

the scalar $c^{\beta \beta }\sigma_{e}^{2}$ gives the variance of ${\hat{\beta }}_{j}$ so the statistic for testing $H_{0}:$ $\beta_{j}=0$ is

$$z_{j}=\frac{{\hat{\beta }}_{j}}{\sigma_{e}\sqrt{c^{\beta \beta }}};\;j=1,2,\ldots .p.$$ (20)

Observe that

$$G_{\left[-j\right]}=G-x_{j}{x^{\prime }}_{j}.$$ (21)

Again using (47) in *Appendix A* produces

$$G_{\left[-j\right]}^{-1}={\left(G-x_{j}{x^{\prime }}_{j}\right)}^{-1}=G^{-1}+\frac{G^{-1}x_{j}{x^{\prime }}_{j}G^{-1}}{1-{x^{\prime }}_{j}G^{-1}x_{j}};j=1,2,\ldots ,p,$$ (22)

and **G** needs to be inverted only once.

As shown earlier, the ${\hat{\beta }}_{j}$ solution in (18) is the same whether or not marker *j* is used when building **G**. It just remains to see whether or not the same holds for BLUP $\left({\hat{g}}_{\left[-j\right]}\right)$. To examine this issue, consider the strong-arm (*i.e.*, without using the mixed model equations) representation of BLUP

$$\hat{g}=\sigma_{g}^{2}GV^{-1}z_{j},$$ (23)

where $z_{j}=y-x_{j}{\hat{\beta }}_{j}$ and

$${\hat{g}}_{\left[-j\right]}=\sigma_{g}^{2}G_{\left[-j\right]}V_{\left[-j\right]}^{-1}z_{j},$$ (24)

where $V_{\left[-j\right]}^{-1}$ is the phenotypic variance-covariance matrix stemming from use of $G_{\left[-j\right]}$ in lieu of **G**. It is shown in *Appendix C* that ${\hat{g}}_{\left[-j\right]}=\hat{g}$ for any j.

It is concluded that point estimates and point predictions from GLS $\left(\beta_{j}\right)$ and BLUP (g), respectively, are invariant with respect to whether or not the marker being tested as a fixed effect is included or removed when constructing the type of genomic relationship matrix used here.

### Generalizations

#### Several markers tested as fixed effects simultaneously:

Expressions (14), (15), and (22) generalize to the situation where *m* markers , instead of a single one, are removed from **X** when forming **G**, and their effects are tested jointly for association. Let $X_{\left[m\text{ out}\right]}$ be a matrix of order n×m whose columns pertain to the markers being tested as fixed effects in an m− marker GWAS, that is, a multiple regression on *m* markers is used. Then

$$V_{\left[m\text{ out}\right]}=V-\sigma_{g}^{2}X_{\left[m\text{ out}\right]}{X^{\prime }}_{\left[m\text{ out}\right]}$$ (25)

and

$$G_{\left[m\text{ out}\right]}=G-X_{\left[m\text{ out}\right]}{X^{\prime }}_{\left[m\text{ out}\right]}.$$ (26)

If the inverses indicated below exist, application of (43) in *Appendix A* gives

$$V_{\left[m\text{ out}\right]}^{-1}=V^{-1}+\sigma_{g}^{2}V^{-1}X_{\left[m\text{ out}\right]}{\left[I-\sigma_{g}^{2}{X^{\prime }}_{\left[m\text{ out}\right]}V^{-1}X_{\left[m\text{ out}\right]}\right]}^{-1}{X^{\prime }}_{\left[m\text{ out}\right]}V^{-1}$$ (27)

and

$$G_{\left[m\text{ out}\right]}^{-1}=G^{-1}+G^{-1}X_{\left[m\text{ out}\right]}{\left[I-{X^{\prime }}_{\left[m\text{ out}\right]}G^{-1}X_{\left[m\text{ out}\right]}\right]}^{-1}{X^{\prime }}_{\left[m\text{ out}\right]}G^{-1}.$$ (28)

For $G_{\left[m\text{ out}\right]}$ to be nonsingular, n≤p−m must hold.

Assuming that variance components remain unaltered if *m* markers are left out in the build-up of G (reasonable for small *m*), the GLS estimator is

$${\hat{\beta }}_{\left[m\;\text{out}\right]}={\left({X^{\prime }}_{\left[m\text{ out}\right]}V_{\left[m\;\text{out}\right]}^{-1}X_{\left[m\;\text{out}\right]}\right)}^{-1}\left({X^{\prime }}_{\left[m\text{ out}\right]}V_{\left[m\;\text{out}\right]}^{-1}y\right).$$ (29)

After algebra

$$\begin{array}{c} {X^{\prime }}_{\left[m\text{ out}\right]}V_{\left[m\;\text{out}\right]}^{-1}\\ ={X^{\prime }}_{\left[m\text{ out}\right]}\left\{V^{-1}+\sigma_{g}^{2}V^{-1}X_{\left[m\;\text{out}\right]}{\left[I-\sigma_{g}^{2}{X^{\prime }}_{\left[m\text{ out}\right]}V^{-1}X_{\left[m\;\text{out}\right]}\right]}^{-1}{X^{\prime }}_{\left[m\text{ out}\right]}V^{-1}\right\}\\ ={\left[I-\sigma_{g}^{2}{X^{\prime }}_{\left[m\text{ out}\right]}V^{-1}X_{\left[m\;\text{out}\right]}\right]}^{-1}{X^{\prime }}_{\left[m\text{ out}\right]}V^{-1} \end{array}$$ (30)

Using the preceding in (29)

$$\begin{array}{c} {\hat{\beta }}_{\left[m\;\text{out}\right]}={\left\{{\left[I-\sigma_{g}^{2}{X^{\prime }}_{\left[m\text{ out}\right]}V^{-1}X_{\left[m\;\text{out}\right]}\right]}^{-1}{X^{\prime }}_{\left[m\text{ out}\right]}V^{-1}X_{\left[m\;\text{out}\right]}\right\}}^{-1}\times \\ \;\;\;\;{\left[I-\sigma_{g}^{2}{X^{\prime }}_{\left[m\text{ out}\right]}V^{-1}X_{\left[m\;\text{out}\right]}\right]}^{-1}{X^{\prime }}_{\left[m\text{ out}\right]}V^{-1}y\\ ={\left({X^{\prime }}_{\left[m\text{ out}\right]}V^{-1}X_{\left[m\;\text{out}\right]}\right)}^{-1}{X^{\prime }}_{\left[m\text{ out}\right]}V^{-1}y. \end{array}$$ (31)

Hence, one retrieves the GLS estimator of the *m* regressions obtained without any modification of the genomic or phenotypic variance-covariance matrices. The variance of the estimator is

$$\begin{array}{l} Var\left({\hat{\beta }}_{\left[m\;\text{out}\right]}\right)\\ ={\left({X^{\prime }}_{\left[m\text{ out}\right]}V^{-1}X_{\left[m\;\text{out}\right]}\right)}^{-1}{X^{\prime }}_{\left[m\text{ out}\right]}V^{-1}V_{\left[m\;\text{out}\right]}V^{-1}X_{\left[m\;\text{out}\right]}{\left({X^{\prime }}_{\left[m\text{ out}\right]}V^{-1}X_{\left[m\;\text{out}\right]}\right)}^{-1}\\ ={\left({X^{\prime }}_{\left[m\text{ out}\right]}V^{-1}X_{\left[m\;\text{out}\right]}\right)}^{-1}-I\sigma_{g}^{2}, \end{array}$$ (32)

where the identity matrix has order m.

#### Removing eigenvectors from G:

The eigen-decomposition of **G** (suppose it is positive-definite) produces

$$G=U\Lambda U^{\prime },$$ (33)

where $U=\left[\begin{array}{cccccc} U_{1} & U_{2} & . & . & . & U_{n} \end{array}\right]$ is the n×n matrix of orthogonal eigenvectors of **G** and $\Lambda =\left\{\lambda_{i}\right\}$ is a diagonal matrix containing the *n* eigenvalues; note that $G=\sum_{i=1}^{n}U_{i}{U^{\prime }}_{i}\lambda_{i}$. Consider the model in (5) and, as in Janss *et al.* (2012), use the equivalent matrix form representation based on putting g=Uα

$$y=x_{j}\beta +U\alpha +e,$$ (34)

where $\alpha ∼N\left(0,\Lambda \sigma_{g}^{2}\right)$ and $\sigma_{g}^{2}$ is the marked genetic variance. The phenotypic variance-covariance matrix is

$$V=U\Lambda U^{\prime }\sigma_{g}^{2}+I\sigma_{e}^{2}=\sum_{i=1}^{n}U_{i}{U^{\prime }}_{i}\lambda_{i}\sigma_{g}^{2}+I\sigma_{e}^{2},$$ (35)

so $\lambda_{i}\sigma_{g}^{2}$ is the genetic variance accounted for by eigenvector *i*.

Population structure is often accounted for by regressing phenotypes on some eigenvectors or on PC of **G**. Suppose that the regressions on the first two eigenvectors are treated as fixed to account for some structure; the SMR model becomes

$$y={\left\{x_{j}\beta +U_{1}\alpha_{1}+U_{2}\alpha_{2}\right\}}_{Fixed}+{\left\{U_{\left[-1-2\right]}\alpha_{\left[-1-2\right]}\right\}}_{Random}+e,$$ (36)

where $U_{\left[-1-2\right]}\alpha_{\left[-1-2\right]}=g_{\left[-1-2\right]}$ is the genetic signal marked by the genomic relationship matrix after removing its first two eigenvectors; $U_{\left[-1-2\right]}$, of order n×(n−2), is U with its first two columns removed, and $\alpha_{\left[-1-2\right]}$ is the corresponding vector of n−2 zero-mean random regression coefficients on $U_{\left[-1-2\right]}$. Above, ${\left\{.\right\}}_{Fixed}$ and ${\left\{.\right\}}_{Random}$ denote the fixed and random terms in the model, respectively.

Let $V_{\left[-1-2\right]}$ be the resulting variance-covariance matrix of **y**, and take the variance components as known, so that

$$\begin{array}{c} Var\left(g_{\left[-1-2\right]}\right)=G_{{}_{\left[-1-2\right]}}=G-\left[\begin{array}{cc} U_{1} & U_{2} \end{array}\right]\left[\begin{array}{cc} \lambda_{1} & 0\\ 0 & \lambda_{2} \end{array}\right]\left[\begin{array}{c} {U^{\prime }}_{1}\\ {U^{\prime }}_{2} \end{array}\right]\\ =\sum_{i=3}^{n}U_{i}{U^{\prime }}_{i}\lambda_{i}, \end{array}$$ (37)

and

$$\begin{array}{c} V_{\left[-1-2\right]}=G\sigma_{g}^{2}+I\sigma_{e}^{2}-\sigma_{g}^{2}\sum_{i=1}^{2}U_{i}{U^{\prime }}_{i}\lambda_{i}\\ =V-\sigma_{g}^{2}\left[\begin{array}{cc} U_{1}\sqrt{\lambda_{1}} & U_{2}\sqrt{\lambda_{2}} \end{array}\right]\left[\begin{array}{c} {U^{\prime }}_{1}\sqrt{\lambda_{1}}\\ {U^{\prime }}_{2}\sqrt{\lambda_{2}} \end{array}\right]. \end{array}$$ (38)

Here, $U_{i}^{\text{*}}=U_{i}\sqrt{\lambda_{i}};$ i=1,2, is a PC vector . Application of (43) in *Appendix A* to (38) produces

$$\begin{array}{c} V_{\left[-1-2\right]}^{-1}=V^{-1}+\sigma_{g}^{2}V^{-1}\left[\begin{array}{cc} U_{1}^{\text{*}} & U_{2}^{\text{*}} \end{array}\right]\\ \;\;\;\;\times {\left\{I-\sigma_{g}^{2}\left[\begin{array}{c} U_{1}^{\text{*}\prime }\\ U_{2}^{\text{*}\prime } \end{array}\right]V^{-1}\left[\begin{array}{cc} U_{1}^{\text{*}} & U_{2}^{\text{*}} \end{array}\right]\right\}}^{-1}\left[\begin{array}{c} U_{1}^{\text{*}\prime }\\ U_{2}^{\text{*}\prime } \end{array}\right]V^{-1}\\ =V^{-1}+\sigma_{g}^{2}V^{-1}\left[\begin{array}{cc} U_{1}^{\text{*}} & U_{2}^{\text{*}} \end{array}\right]\\ \;\;\;\;\times {\left[\begin{array}{cc} 1-\sigma_{g}^{2}U_{1}^{\text{*}\prime }V^{-1}U_{1}^{\text{*}} & -\sigma_{g}^{2}U_{1}^{\text{*}\prime }V^{-1}U_{2}^{\text{*}}\\ -\sigma_{g}^{2}U_{2}^{\text{*}\prime }V^{-1}U_{1}^{\text{*}} & 1-\sigma_{g}^{2}U_{2}^{\text{*}\prime }V^{-1}U_{2}^{\text{*}} \end{array}\right]}^{-1}\left[\begin{array}{c} U_{1}^{\text{*}\prime }\\ U_{2}^{\text{*}\prime } \end{array}\right]V^{-1}. \end{array}$$ (39)

Once $V_{\left[-1-2\right]}^{-1}$ is formed, (15) can be employed to obtain

$$V_{\left[-1-2-j\right]}^{-1}=V_{\left[-1-2\right]}^{-1}\left[I+\frac{\sigma_{g}^{2}}{1-\sigma_{g}^{2}{x^{\prime }}_{j}{t^{\prime }}_{j}^{\#}}x_{j}{t^{\prime }}_{j}^{\#}\right];\;j=1,2,\ldots ,p,$$ (40)

where ${t^{\prime }}_{j}^{\#}={x^{\prime }}_{j}V_{\left[-1-2\right]}^{-1}.$ Instead of inverting *p* phenotypic variance-covariance matrices, one extracts eigenvectors from G and inverts $V_{\left[-1-2\right]}$ only once. In this situation, model (36) can be written as

$$y=W\theta +g_{\left[-1-2\right]}+e,$$ (41)

where $W_{n\times 3}=\left[\begin{array}{ccc} x_{j} & U_{1} & U_{2} \end{array}\right]$ and $\theta^{\prime }=\left[\begin{array}{ccc} \beta & \alpha_{1} & \alpha_{2} \end{array}\right].$ The GLS estimator of the three regression coefficients is

$$\hat{\theta }={\left(W^{\prime }V_{\left[-1-2-j\right]}^{-1}W\right)}^{-1}W^{\prime }V_{\left[-1-2-j\right]}^{-1}y.$$ (42)

Using a wheat data set described later, we set $\sigma_{g}^{2}=\sigma_{e}^{2}=1$ and calculated GLS estimates of the regressions on each of the first five markers, using $V,V_{\left[-1\right]},V_{\left[-1-2\right]},V_{\left[-1-2-3\right]}$, and $V_{\left[-1-2-3-4\right]}$ where the subscripts denote the eigenvectors removed. The estimates were

$$\begin{array}{c} {\hat{\beta }}_{j}=-0.2563,\;0.6901,\;0.0231,\;-0.3036,\;0.2414;\\ {\hat{\beta }}_{j\left[-1\right]}=-0.2567,\;0.6934,\;0.0231,\;-0.3055,\;0.2427,\\ {\hat{\beta }}_{j\left[-1-2\right]}=-0.2572,\;0.6933,\;0.0229,\;-0.3061,\;0.2424,\\ {\hat{\beta }}_{j\left[-1-2-3\right]}=-0.2571,\;0.6934,\;0.0231,\;-0.3061,\;0.2427,\\ {\hat{\beta }}_{j\left[-1-2-3-4\right]}=-0.2586,\;0.6934,\;0.0231,\;-0.3072,\;0.2426. \end{array}$$

Differences were minor, and corresponding BLUPs were very similar as well. For example, using marker 3, the regression of $BLUP_{\left[3,-1-2-3-4\right]}$ on $BLUP_{\left[3\right]}$ had 0.009 as intercept and 0.9951 as slope. Removing eigenvectors makes a difference, but it had a negligible practical importance in this example.

## Case Studies with Wheat and Arabidopsis Data

### Wheat

A publicly available wheat data set was employed to investigate several issues associated with removing markers or eigenvectors from G, including impact on maximum likelihood estimates of variance components. The wheat data were downloaded from package BGLR (Pérez and de los Campos 2014); these data have also been used by, *e.g.*, Crossa *et al.* (2010), Gianola *et al.* (2011) and Long *et al.* (2011). The data originated from several international trials conducted at the International Maize and Wheat Improvement Center (CIMMYT), Mexico. There are 599 wheat inbred lines, each genotyped with 1279 DArT (Diversity Array Technology) markers and planted in four environments. The target trait was yield in environment 1. Here n=599 and p=1279. The DArT markers are binary (0,1) denoting presence or absence of an allele at a marker locus in a given line. In this data set there is no information on chromosomal location of markers, but this does not hamper illustration of concepts.

### Arabidopsis

We also used the *A. thaliana* data set described by Atwell *et al.* (2010) and Wimmer *et al.* (2013), mainly for illustrating the impacts of eigenvector removal on inferences. Norborg *et al.* (2005) and Atwell *et al.* (2010) pointed out that this sample of accessions suggests a complex structure in the population, making the data interesting for our purposes. The data, available in the R Synbreed package (Wimmer *et al.* 2012), represents 199 accessions genotyped with a custom Affymetrix 250K SNP chip, and measured for a number of phenotypes. As in Wimmer *et al.* (2013), flowering time (n=194), plant diameter (n=180), and FRIGIDA (n=164) gene expression were chosen as target phenotyes; marker genotypes are pre-edited in the package, and 215,947 SNP loci were used in the analysis.

### GWAS: OLS *vs.* GLS analyses

We compared SMR-OLS *vs.* GLS in the wheat data at two specified values of the variance ratio or, equivalently, of genomic heritability. Marker genotypes were centered to have a mean of zero, marker by marker, for all 1279 DArT polymorphisms; phenotypes were already standardized to have a null mean and variance 1. For OLS, computation was done using the lm function available in the R package (http://www.r-project.org/). In GLS, the genomic relationship matrix used was as follows: 1) with X being the matrix of centered markers, we formed $G=\text{XX′}/\left(p\bar{d}\right)=\left\{g_{ij}\right\}$; here, $\bar{d}$ is the mean of the diagonal values of XX′/p and $g_{ij}$ measures similarity in state between individuals *i* and *j*. 2) We then formed $V^{\text{*}}=G\frac{h_{g}^{2}}{1-h_{g}^{2}}+I$, and, for the purpose of examining sensitivity, set genomic heritability in the GLS analysis to $h_{g}^{2}=\left(0.10,0.25\right),$ representing an increase in the signal to noise ratio when going from 0.10 to 0.25. GLS was implemented using the lm function via transformation of the phenotypes and of the marker incidence matrix, as shown in *Appendix D*.

The SMR OLS and GLS analyses gave similar inferences in terms of regression coefficients, $R^{2}$ (percentage of corrected sums of squares of grain yield explained by the model), and p− values, but the GLS residual variances were smaller. While 29 markers were found significant (Bonferroni corrected *p* values) for OLS, the GLS analyses at $h^{2}=0.10$ and 0.25 produced 32 significances (31 in common). As is typically the case for quantitative traits such as grain yield, most single marker based models explained a small fraction of the variation: the largest $R^{2}$ observed were 7.27%, 7.28%, and 7.29% for OLS, GLS(0.10), and GLS(0.25), respectively. Here, $R^{2}$ was the standard measure used in OLS and GLS (using *Appendix D*, one can calculate the GLS statistics employing OLS computations); alternative measures are discussed by Sun *et al.* (2010).

The GWAS literature does not emphasize enough that the explanatory power of a model and estimates of effect sizes change markedly when additional markers are included in the specification, *i.e.*, failure to account for other variants is one of the most obvious explanations of missing heritability (Maher 2008) in SMR. Adding up $R^{2}$ from SMR gives a distorted picture of the variability explained, because LD is ignored (*e.g.*, Gianola *et al.* 2013). To illustrate how effect size in GWAS was affected by model specification, we fitted jointly by least-squares multiple marker regression (MMR) all 29 markers found significant in OLS SMR. The $R^{2}$ of this model was 28.3%. In this MMR, however, only two markers were significant at $\alpha =0.05/29=1.72\times 10^{-3}$ (Bonferroni correction); these *p* values are of course incorrect in a sequential approach such as the one followed here. Effect size estimates were different, including sign changes (some markers with a negative SMR estimate became positive in MMR, and vice versa) The MMR estimates were larger in size and more variable (SE not shown) due to the colinearity caused by strong LD between some markers. In theory, SMR may have a larger bias (relative to causal loci in a multifactorial model) than MMR, but the latter produces estimates with more variability. The mean-squared error of estimation cannot be evaluated in the absence of knowledge of model parameters; the true marker effect depends on the effects of the QTL affecting the trait, and on the unknown LD relationships between markers and QTL, these being of a multivariate nature in the case of complex traits (de los Campos *et al.* 2015)

### Effect of removing a single marker from G on genomic heritability

**Figure 1:**
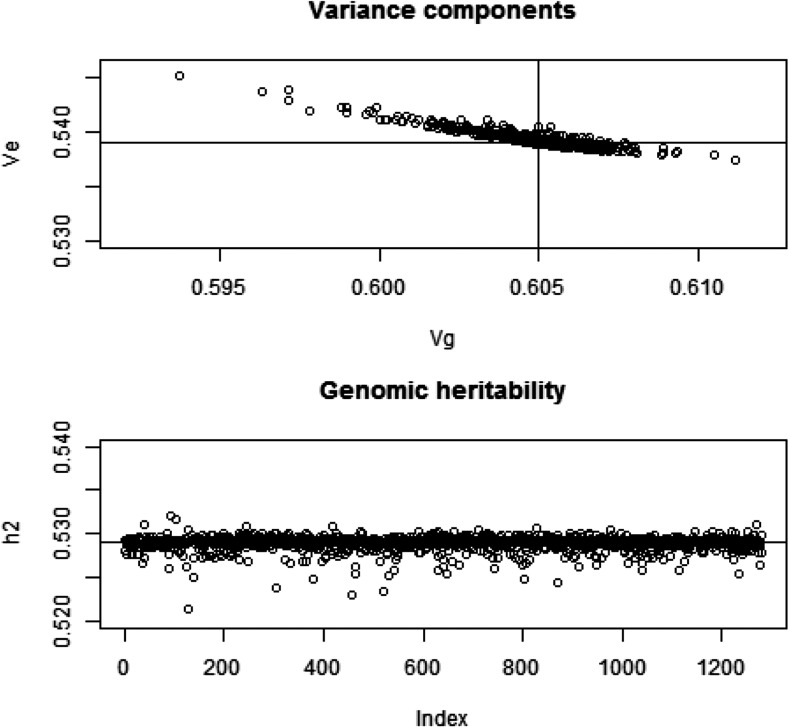
Wheat: maximum likelihood (ML) estimates of genomic (Vg) and residual (Ve) variance components and of genomic heritability (h2) corresponding to 1279 models with markers removed, one at a time, when forming the genomic relationship matrix (**G**). Top panel: variance components; horizontal and vertical lines indicate ML estimates with all markers in **G**. Bottom panel: genomic heritability; horizontal line indicates the estimate with all markers.

Since our analytical developments assume that marker removal does not affect the partition of variance, we measured the extent to which this assumption held using the wheat data set. The likelihood was formed under $y∼N\left(0,G\sigma_{g}^{2}+I\sigma_{e}^{2}\right)$. Here, we took $G=\text{XX′}/\left(\bar{d}p\right)$, where $\bar{d}$ is the mean of the diagonal elements of $XX^{\prime }$ (markers were centered) and estimated the two variance components by maximum likelihood using an eigen-decomposition algorithm of $G\sigma_{g}^{2}+I\sigma_{e}^{2}$ (*e.g.*, Janss *et al.* 2012) that renders computation fast. Estimates obtained with all markers included in G were ${\tilde{\sigma }}_{g}^{2}=0.605\pm 0.102$ and ${\tilde{\sigma }}_{e}^{2}=0.539\pm 0.04$; genomic heritability was 0.529. Convergence was assessed and confirmed by beginning the iteration from different sets of starting values. Further, we constructed 1279 genomic relationship matrices by excluding one marker at a time, *i.e.*, $G_{\left[-j\right]}$, j=1,2,…,1279; convergence was assumed for each case, starting the iteration using a value of 0.5 for each of the two variance components. The estimates obtained are shown in Figure 1; larger estimates of genomic variance were associated with smaller estimates of residual variance. Departure of parameter estimates from the values obtained when all markers entered into **G** was very mild for all markers. Out of the 1279 sets of estimates of genomic and residual variance, 797 were smaller than ${\tilde{\sigma }}_{g}^{2}$ due to marker exclusion.

**Figure 2:**
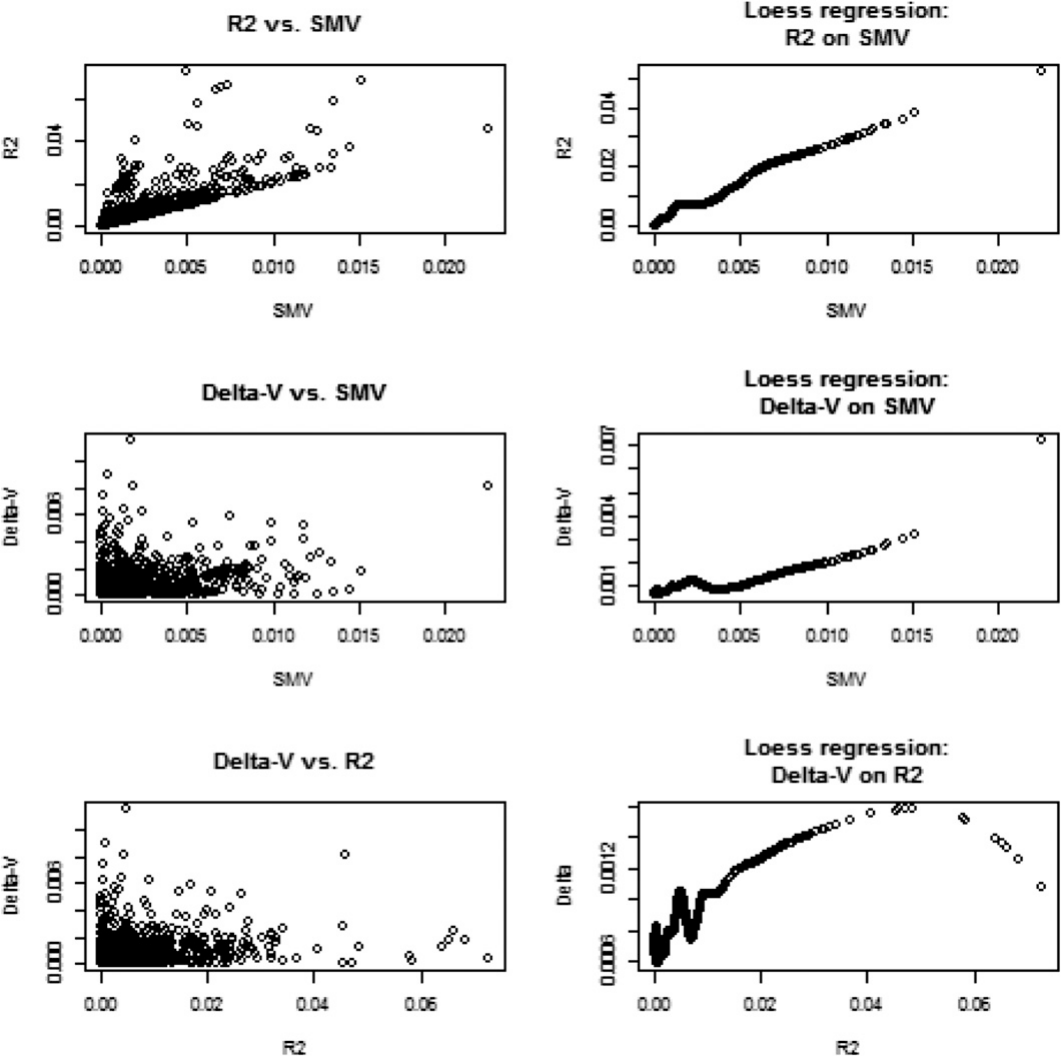
Wheat: associations between change in absolute value of genomic variance estimate due to removal of a marker (Δ‐V), and the R2 and marker variance assessment (SMV) from single marker regression. Right panels show fitted values of a local regression (LOESS) with span parameter = 0.25.

**Figure 3:**
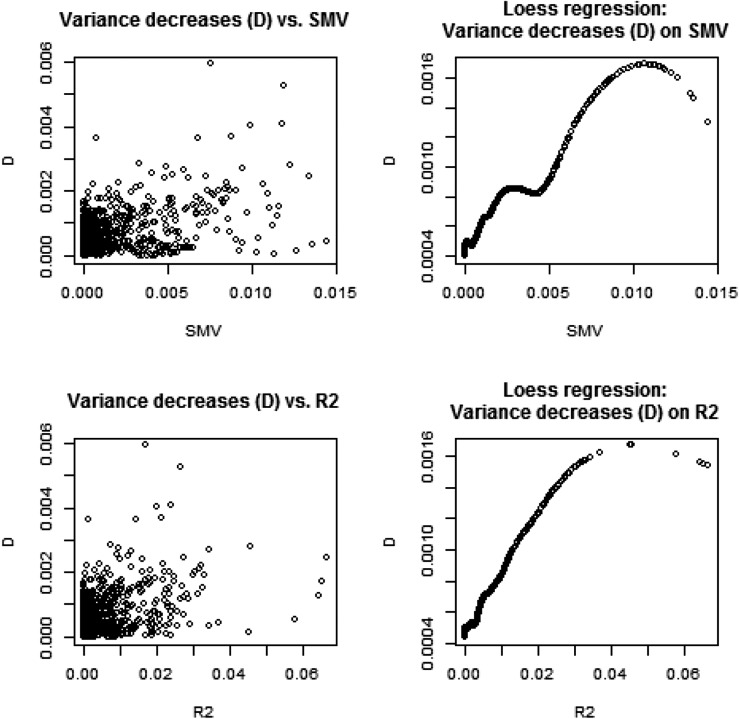
Wheat: associations between decrease (D) in genomic variance estimate due to removal of a marker from the genomic relationship matrix (**G**), and the R2 and marker variance assessment (SMV) from single marker regression. Plot depicts the 482 cases where marker removal reduced the genomic variance estimate relative to the estimate obtained with all markers contributing to **G**. LOESS span parameter = 0.40.

We assessed genetic variability as often done in GWAS via SMR, and related the corresponding metrics to what would be suggested by a variance component analysis. In a SMR, the contribution of marker *j* to variability is calculated as $SMV=2q\left(1-q\right){\tilde{\beta }}^{2}$ where $\tilde{\beta }$ is typically an OLS estimate and *q* is allelic frequency; SMV stands for single marker variance. This formula is very crude because it assumes that the trait is mono-factorial, or that loci are in linkage equilibrium, so the total genetic variance would be the sum of variances contributed by each of the loci (*e.g.*, Gianola *et al.* 2009, 2013). For inbred lines such as in the wheat data, the metric is $q\left(1-q\right){\tilde{\beta }}^{2}.$ We evaluated if changes in estimates of $\sigma_{g}^{2}$ due to removing marker *j* from the **G** matrix correlated with $q_{j}\left(1-q_{j}\right){\tilde{\beta }}_{\text{OLS},j}^{2}$, and with standard $R_{j}^{2}$ values from SMR. Since marker removal sometimes increased, sometimes decreased, the variance estimates relative to ${\tilde{\sigma }}_{g}^{2}=0.605,$ we computed the absolute values of ${\tilde{\sigma }}_{g}^{2}-{\tilde{\sigma }}_{g[-j]}^{2}$; j=1,2,….,1279. Figure 2 displays relationships between $SMV_{j}$, $R_{j}^{2}$ and $\Delta \left(V\right)=\left|{\tilde{\sigma }}_{g}^{2}-{\tilde{\sigma }}_{g\left[-j\right]}^{2}\right|$. SMV and $R^{2}$ had a clear association; this was expected because $R^{2}$ is proportional to ${\tilde{\beta }}^{2}$ in simple linear regression. Since the relationship between Δ-V and SMV, or $R^{2}$, was less transparent, we extracted a pattern using local regression (LOESS) with a span parameter of 0.25, meaning that a local neighborhood had 320 members (Cleveland 1979). There was a tendency for Δ-V to increase when SMV (or $R^{2})$ increased. This is reasonable because the more variance a marker captures, the larger the decrease from ${\tilde{\sigma }}_{g}^{2}$ should be when such marker is removed from **G**. Since we were unable to monitor convergence for the 1279 sets of estimates, it may be that removing a marker increased ${\tilde{\sigma }}_{g[-j]}^{2}$ relative to ${\tilde{\sigma }}_{g}^{2}$; this phenomenon could be due to convergence to a local maximum and our estimation procedure did not constrain each ${\tilde{\sigma }}_{g[-j]}^{2}$ to be, at most, ${\tilde{\sigma }}_{g}^{2}.$ Thus, we turned attention to the subset of estimates where marker removal reduced the marked additive genetic variance relative to ${\tilde{\sigma }}_{g}^{2}$. This analysis is displayed in Figure 3 and the picture was clear: removing markers from **G** assessed via SMR as making a larger contribution to the variance of the trait did reduce estimates of genomic variance. The impact was very small but detectable.

It is concluded from the preceding that, if the contribution of a marker to **G** is removed, it is safe (especially with dense chips) to use variance components estimated from an all-markers analysis, unless there are some huge effect variants that would probably be detected anyhow. Since we showed analytically that the GLS estimator is invariant with respect to removing markers from **G**, it is unnecessary to re-estimate variance parameters, or to modify **G** at any pass of a SMR GWAS.

### Effect of removing principal components from G on genomic heritability

#### Multi-dimensional scaling of wheat and *Arabidopsis*:

Many GWAS use a SMR model with one or a few regressions on PC of XX′ as fixed effects, to account for population structure. If such an analysis is based on a mixed model with $G\sigma_{g}^{2}$ as genomic covariance structure, the implication is that the PC fitted (with a fixed regression) is not removed from **G**, which is contradictory. To guide the specification of the GWAS model, we searched for genomic structure in the wheat and *Arabidopsis* genotype matrices using multi-dimensional scaling (MDS).

MDS was developed by Kruskal (1964 a, b) to obtain spatial representations of objects in a perceptual K− dimensional topology. A description is in Borg and Groenen (2005), and an application to quantitative genomics is in Zhu and Yu (2009). MDS inputs can be squared Euclidean distances between objects, in our case the p− dimensional genotypes of the 599 wheat lines or the 199 *Arabidopsis* accessions. There were 179,101 and 19,701 Euclidean distances between rows of **X** (or of **G**) in the wheat and *Arabidopsis* data sets, respectively. In MDS, distances are rotated into a matrix whose eigen-decomposition yields the *K*-dimensional coordinates, while preserving distances in some best fit sense. There are two types of MDS: classical and nonmetric. In classical MDS, differences and ratios between distances are preserved. In nonmetric scaling, only the order of the distances is relevant. The best fitting *K* is found at the eigenvalue in which an elbow of an eigenvalue decay plot is observed, or can be derived from a metric called STRESS. Here, squared differences between observed and fitted distances are summed over the two dimensions, and expressed relative to the sum of all observed squared distances. If STRESS (the square root of the preceding quantity) is smaller than 5–10%, the corresponding dimension is deemed to give a satisfactory fit (Kruskal, 1964b).

**Figure 4:**
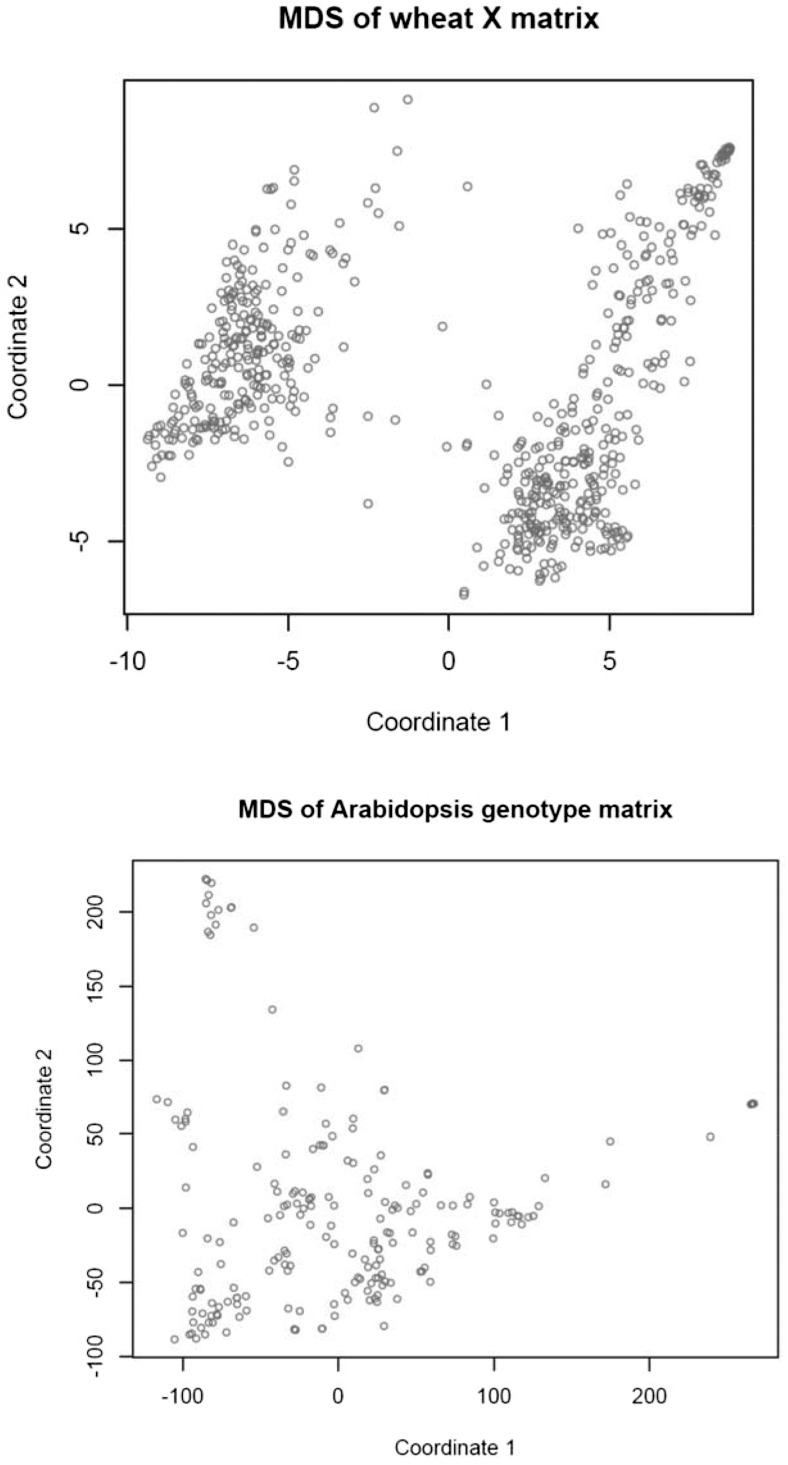
Multidimensional scaling of SNP genotype matrices in the wheat and *Arabidopsis* data sets: first two dimensions.

**Figure 5:**
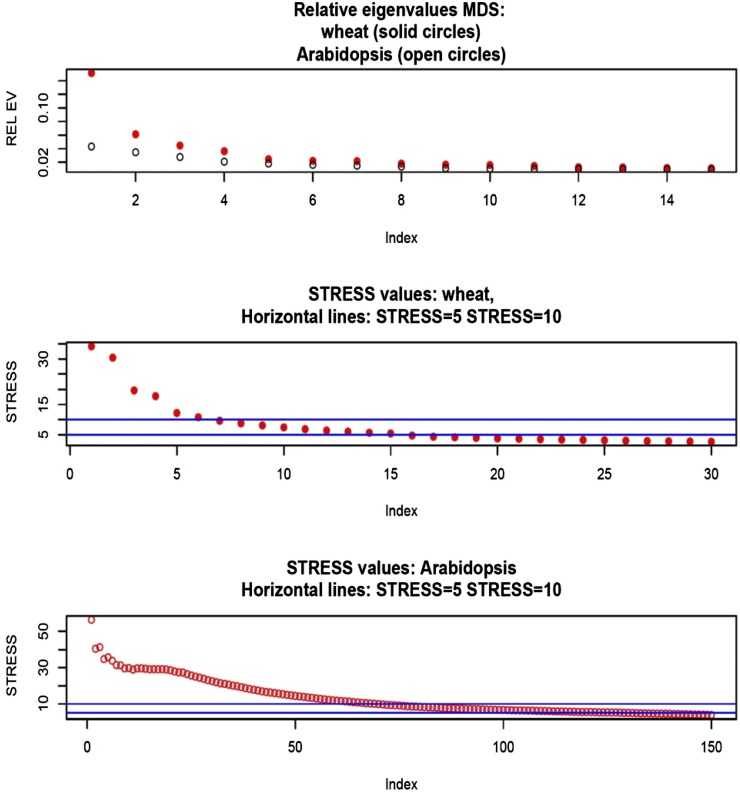
Multidimensional scaling of SNP genotype matrices in the wheat and *Arabidopsis* data sets. Top panel: eigenvalue (relative to their sum) decay. Middle panel: STRESS metric in wheat. Bottom panel: STRESS metric in Arabidopsis.

We fed the wheat and *Arabidopsis* distances to the R functions cmdscale and ISOmds, fitted models with K=1,2,…,198 (*Arabidopsis*) and K=1,2,…,30 (wheat) dimensions, and calculated STRESS for each model. Spatial representations obtained for models with two dimensions are in Figure 4: the perception of structure is much clearer in wheat than in *Arabidopsis*. In wheat, the first coordinate separates lines into two well delineated groups; the second coordinate stretches the lines within groups along the *y*-axis. A two-dimensional representation seemed insufficient in the *Arabidopsis* data. Figure 5 (top panel) presents a scree plot of eigenvalues expressed as a fraction of the sum of all MDS eigenvalues: the first five eigenvalues represented 31.8% and 14.5% of the variation in wheat and *Arabidopsis*, respectively; save for the first two, there were no clearly dominant values in *Arabidopsis*. The middle and bottom panels show STRESS (nonmetric MDS) for models of different dimensionality. In the wheat data set, a satisfactory fit (STRESS = 5–10%; Kruskal 1964b) was obtained with K=5−10 dimensions, but at least 70 dimensions were needed to fit the *Arabidopsis* distances reasonably well. The implication is that population structure in wheat may not be accounted properly with a single PC. In the *Arabidopsis* collection, the topology was less sharp, suggesting an aggregation similar to the family structure typically encountered in animal breeding or in humans (see Supplementary Figure 4 in Atwell *et al.* 2010). If the latter is the case, use of a kinship matrix in the GWAS may suffice, without the need of fitting principal components as fixed effects. In wheat, on the other hand, a kinship matrix would account for similarity among lines, but not for differences in mean among the few strata suggested by Figure 4 and Figure 5.

#### PC and variance components:

**Figure 6:**
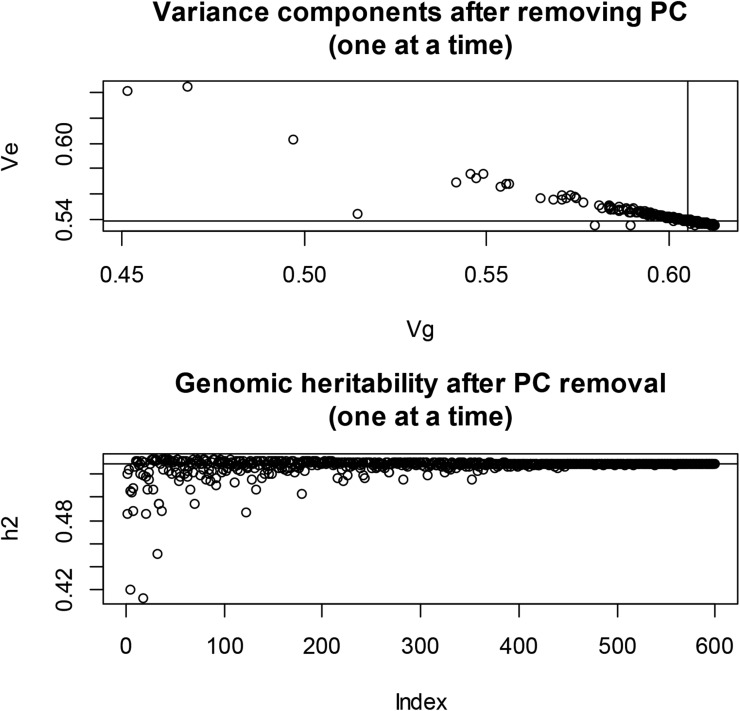
Wheat: maximum likelihood estimates of genomic (Vg) and residual (Ve) variance components and of genomic heritability (h2) corresponding to 599 models with principal components (PC) removed, one at a time, when forming the genomic relationship matrix (**G**). Top panel: variance components. Bottom panel: genomic heritability. Horizontal and vertical lines indicate estimates found with all PC in **G**.

**Figure 7:**
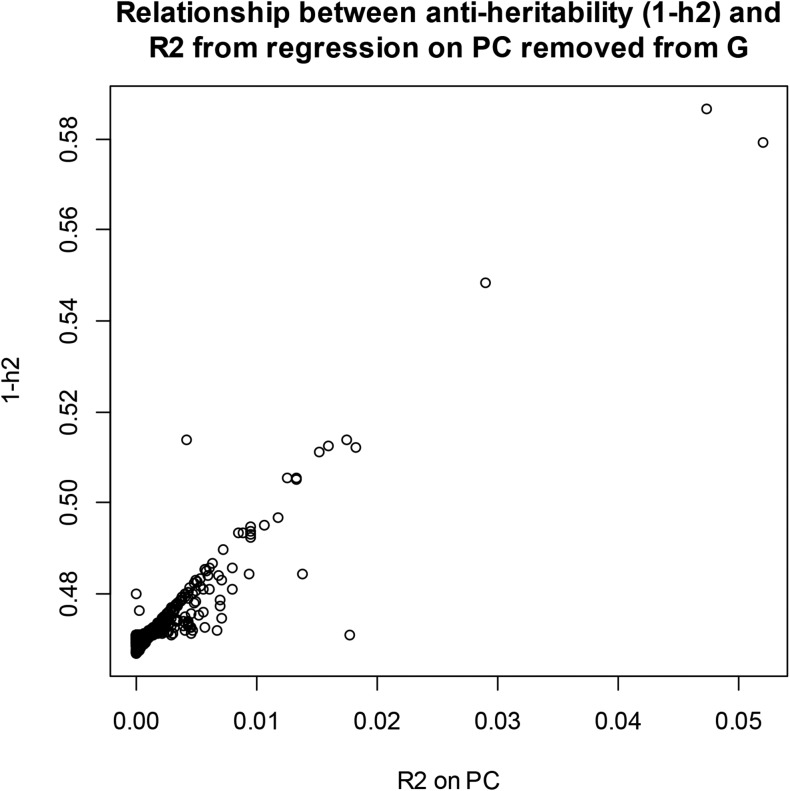
Wheat: relationship between genomic antiheritability (1–h2) after removing each one of the PC of **G** and R2 from the ordinary least‐squares (OLS) regression of yield on each of the PC.

We examined the effect of removing PC from G on maximum likelihood estimates of the two variance components. In wheat, after extracting the 599 PC of **G**, maximum likelihood estimates of $\sigma_{e}^{2}$ and $\sigma_{g}^{2}$ were obtained by removing one PC at each time when building the genomic relationship matrix. The impact on the estimates was noticeable (Figure 6): removing “dominant” PC from G produced much lower estimates of genomic variance and of genomic heritability than when all PCs entered into **G** (recall that genomic variance and heritability of yield were 0.605 and 0.529, respectively), and larger estimates of residual variance. The relationship between $1-h_{g}^{2}$ (genomic antiheritability) and the $R^{2}$ from the OLS regression of grain yield on each of the PC is shown in Figure 7: removal of PC with the largest $R^{2}$ resulted in the largest antiheritability estimates. PC with the strongest association with the trait also had the largest impact on $h^{2}$ estimates when removed from **G** (not shown) In short, if a PC is treated as fixed but not removed from **G**, the residual variance will be understated, because the fact that such PC also contributes to genomic variance would not be accounted for properly. Genomic heritability should be re-estimated if PCs are removed from **G**.

**Figure 8:**
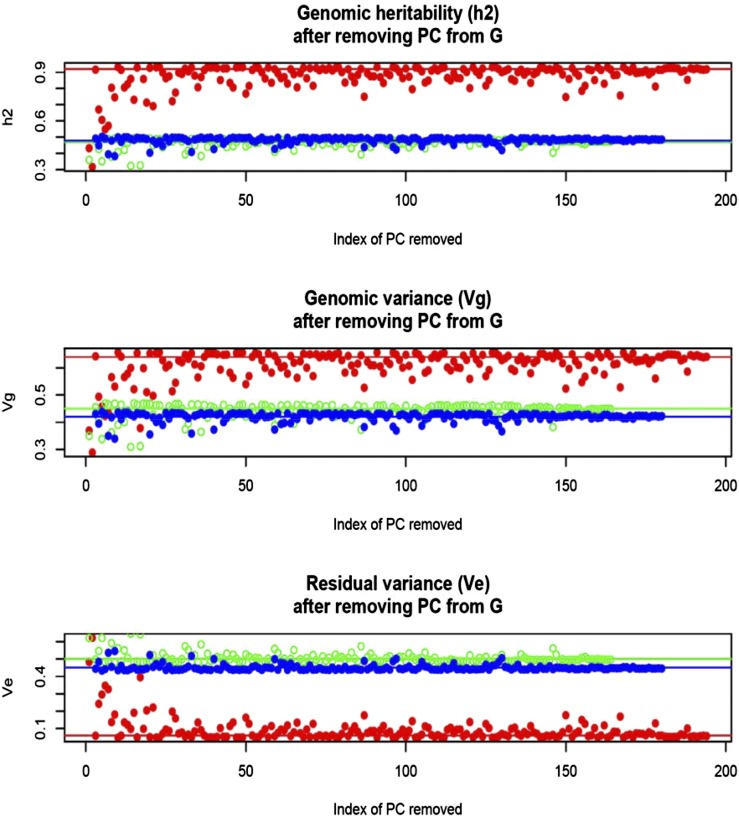
*Arabidopsis*: maximum likelihood estimates of genomic (Vg) and residual (Ve) variance components and of genomic heritability (h2) corresponding to models with PC removed, one at a time, when forming the genomic relationship matrix (**G**). Red: flowering time. Green: FRIGIDA expression. Blue: plant diameter. Horizontal and lines indicate estimates found with all PC in **G**.

In *Arabidopsis*, estimates of variance components and of genomic heritability were $\sigma_{g}^{2}=0.64$, $\sigma_{e}^{2}=0.06,$ and $h_{g}^{2}=0.92$ (flowering time); $\sigma_{g}^{2}=0.45$, $\sigma_{e}^{2}=0.50,$ and $h_{g}^{2}=0.47$ (FRIGIDA), and $\sigma_{g}^{2}=0.42$, $\sigma_{e}^{2}=0.45,$ and $h_{g}^{2}=0.49$ (plant diameter). Close to 50% (FRIGIDA and diameter) and near 100% (flowering time) of the variance among accessions was accounted for by the 215,947 markers used for building the genomic relationship matrices. However, SE of the estimates were very large, reflecting the small number of accessions in the sample. Nevertheless, the heritability estimate of flowering time suggests a large degree of genetic control of the trait. Figure 8 displays effects on the dispersion parameters of removing PC from the genomic relationship matrix. In general, removing any of the first 10–30 PC had the largest impact on the decrease of genomic variance and heritability, and the concomitant increase in residual variance, particularly for flowering time. There were exceptions, however; for instance, removing PC 4 had a similar effect on the decrease of genomic variance than removing PC 100 or PC 110. A larger sample size would probably produce a more discernible pattern.

#### PC in the regression model:

We evaluated the extent to which associations detected by OLS or GLS were affected by accounting for structure, and by whether or not the PC used as regressor was kept as a part of, or excluded from, the genomic relationship matrix **G**. With that objective, we compared estimates from various analyses of the wheat data: 1) OLS on markers with and without the first PC as a covariate; 2) GLS (using maximum likelihood estimates of variance components) on markers with or without the first PC as covariate; 3) GLS as in (2) but with or without the first PC removed from the **G** matrix. As expected, $R^{2}$ increased, while some regressions near 0 became more negative and some more positive when the PC was included in the model; this happened both in OLS and GLS. Several regressions on markers became more significant because the residual variance decreased relative to the one produced by the model without the PC as regressor. Including or excluding the first PC when forming the relationship matrix **G** had a negligible impact on inference, as the metrics used in the comparison aligned on a 45° degree line (results not shown).

**Figure 9:**
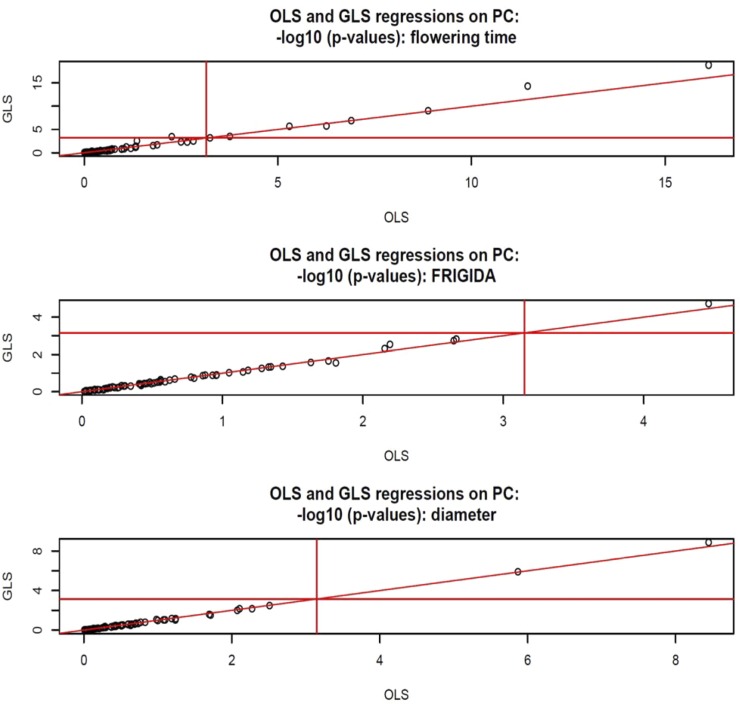
*Arabidopsis*: OLS and generalized least‐squares statistical support for association with 70 principal components of **G** fitted jointly, –log (*p*‐*values*, base 10), for flowering time, FRIGIDA expression, and plant diameter. Horizontal and vertical lines are at 3.15, corresponding to a Bonferroni correction for 70 comparisons with single test significance at 5%.

In *Arabidopsis*, we fitted a multiple-regression on the first 70 PC; this was done by OLS and by GLS (maximum likelihood estimates of variance components, all PC in **G**). The support for statistical significance is shown in Figure 9. For flowering time, nine (eight) of the 70 regressions were declared significant by GLS (OLS). For FRIGIDA, only one regression was deemed significant by GLS, and none for OLS. For plant diameter, the two methods agreed. The analyses illustrate that the effects of population structure are trait-dependent. For flowering time, the trait with the largest relative amount of genetic variance, several PC seem needed for an appropriate GWAS; ignoring this complex structure could provide a false idea of association expected within a homogeneous group of accessions.

**Figure 10:**
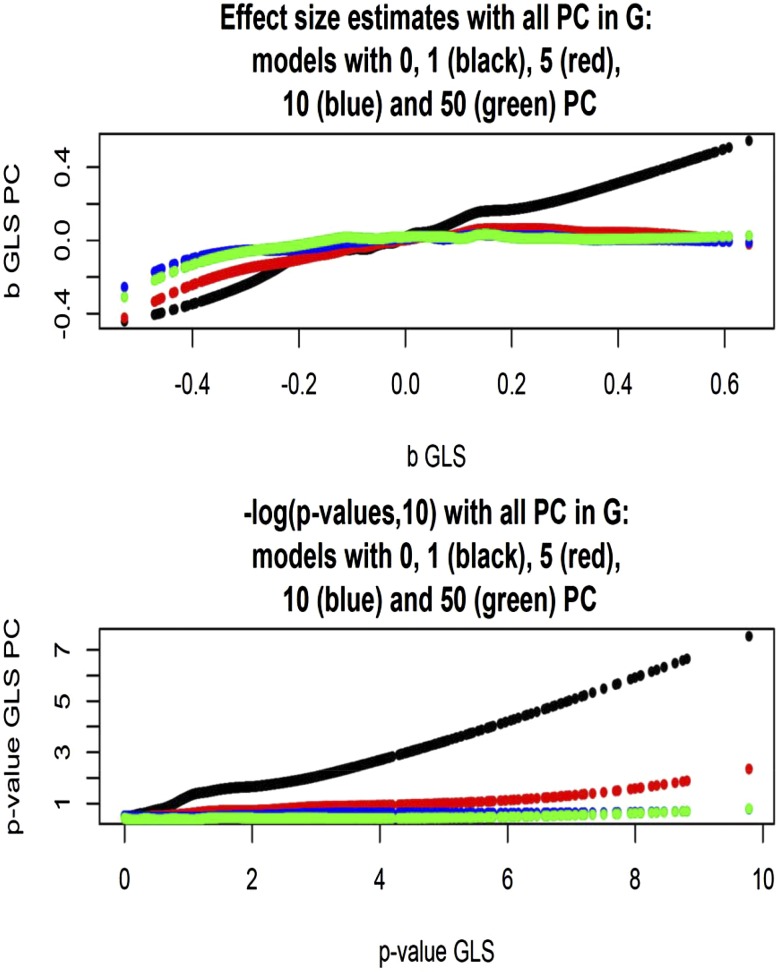
*Arabidopsis*: effect sizes and statistical support for association between flowering time and 5000 markers (chromosome 2) for models without or with one, five, 10, or 50 PC as fixed covariates. Models use a genomic relationship matrix with all its PC and corresponding maximum likelihood estimates of variance components. Scatter was smoothed using LOESS.

We extracted the first 5000 markers from *Arabidopsis* chromosome 2, and used flowering time (the trait with 90% heritability) as a target trait for evaluating alternative GWAS model specifications. Genomic relationship matrices were constructed with all 215,947 markers, and the regression model included the single marker tested, and either zero, one, five, 10, or 50 PC as fixed covariates. Figure 10 gives a plot of allelic substitution effects, and of $-{\text{log}}_{10}\left(p-value\right)$: the *x*-axis labels effect size estimates (top panel) and statistical support values (bottom panel) for the model without PC in the regression structure. Clearly, accounting for structure had a marked effect on estimates of marker effects, and on statistical support: as the number of PC in the regression increased, effect sizes decreased in absolute value and support for association vanished. While a large number of markers would be declared as associated when population structure is ignored, only a few of these would remain significant after the first PC is fitted; none would be significant if five or more PC are fitted. When the first PC was removed from **G**, heritability of flowering time dropped from 0.92 to 0.43. When five, 10 or 50 PC were removed, $h^{2}$ decreased further to 0.07, $3.9\times 10^{-6}$ and $1.9\times 10^{-9}.$ It is interesting to note that, while the nonmetric MDS suggested that about 50 dimensions were needed to account for genomic dissimilarity among accessions, only a few dimensions capture the association with trait variance.

**Figure 11:**
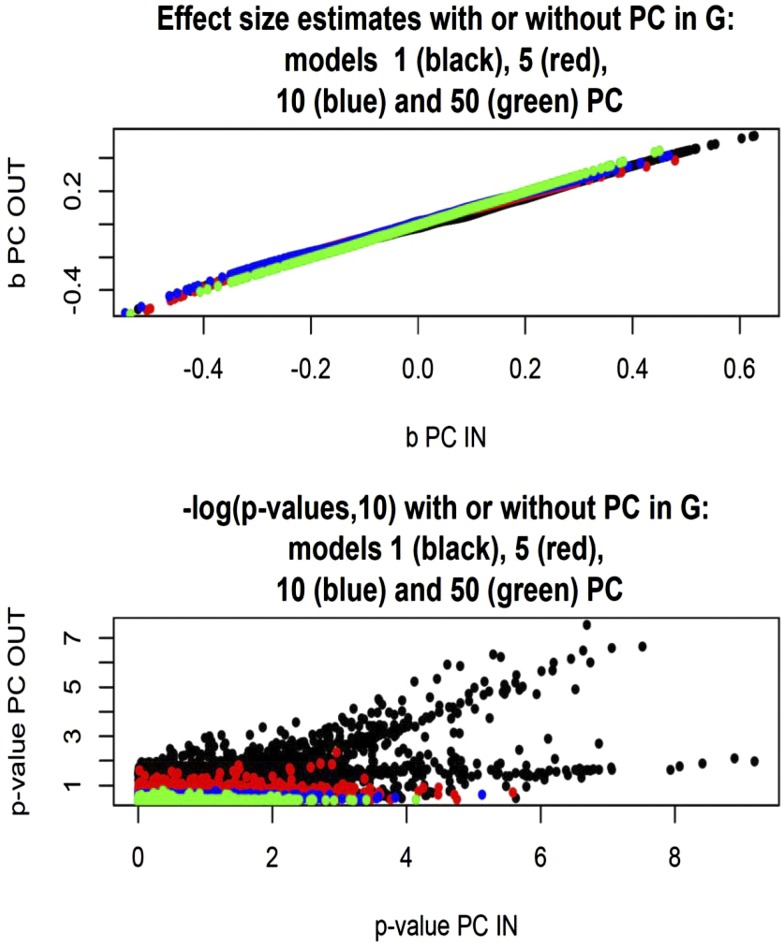
*Arabidopsis*: effect sizes and statistical support [–log(*p*‐*value*, 10)] for association between flowering time and 5000 markers (chromosome 2) for models without or with one, five, 10, or 50 PC tested as fixed covariates. Models use a genomic relationship matrix with or without the PC tested included, and corresponding maximum likelihood estimates of variance components.

Finally, effect size estimates and statistical support were compared for the model in which **G** was left intact when one, five, 10, or 50 PC were fitted in the regression structure, *vs.* the corresponding models with **G** and variance components appropriately modified. As shown in Figure 11 (top panel), effect size estimates aligned well for the two classes of model, but their absolute values were somewhat smaller when the contribution to **G** of the PC tested was taken into consideration. The bottom panel of Figure 11 shows that the statistical support for association essentially vanished when more than two dimensions of the population structure were accounted for via PC.

### Data availability

The authors state that all data necessary for confirming the conclusions presented in the article are represented fully within the article.

## Conclusions

Our study addressed some standing issues in standard GWAS methodology for complex traits, as practiced in animal, human, and plant genetics. We examined the question of how removal of one or more markers from the genomic relationship matrix affects the generalized least-squares estimator (maximum likelihood under normality, and known genomic heritability) of allelic substitution effects in a SMR. It was shown analytically that, if variance components are kept constant, the GLS estimator and the GBLUP predictor of marker additive genetic values are invariant with respect to whether or not the marker(s) tested for association is(are) included when constructing **G**. We also examined the impact of removing PC from **G**, and found that it does matter, and importantly so. Further, and unsurprisingly, estimates of genomic and residual variances were found to be sensitive with respect to the structure of **G**. Concepts were illustrated using publicly available wheat and *Arabidopsis* data sets.

In conclusion, in a homogeneous population, inferences from a GWAS using GLS where the genomic relationship matrix is constructed using all markers does not present clear pitfalls other than the inability of a SMR model to represent the statistical genetic architecture of a complex trait properly. On the other hand, if one or more PC are used as fixed regressors to account for population stratification, the genomic relationship matrix perhaps should be modified, and variance components re-estimated accordingly. In the absence of knowledge of the state of nature, it is impossible to answer unambiguously the question of which approach is best. It has been argued and shown that statistical significance and predictive ability are not synonymous (Lo *et al.* 2015), so perhaps cross-validation could be used for comparing models. An unfortunate duality is that predictive performance does not necessarily provide a guide for explanation (Shmueli 2010).

## Supplementary Material

Supplemental Material

## Appendix A: Sherman-Morrison-Woodbury Formula

Assuming that the inverse matrices involved below exist (*e.g.*, Seber and Lee 2003)

$${\left(A+UBV\right)}^{-1}=A^{-1}-A^{-1}UB{\left(B+BVA^{-1}UB\right)}^{-1}BVA^{-1}.$$ (43)

For the special case B=I, U=±u, and $V=v^{\prime }$

$${\left(A+\text{uv′}\right)}^{-1}=A^{-1}-A^{-1}u{\left(1+v^{\prime }A^{-1}u\right)}^{-1}v^{\prime }A^{-1},$$ (44)

$${\left(A-\text{uv′}\right)}^{-1}=A^{-1}+A^{-1}u{\left(1-v^{\prime }A^{-1}u\right)}^{-1}v^{\prime }A^{-1}.$$ (45)

Since $1-v^{\prime }A^{-1}u$ is scalar

$${\left(A+\text{uv′}\right)}^{-1}=A^{-1}-\frac{A^{-1}uv^{\prime }A^{-1}}{1+v^{\prime }A^{-1}u}$$ (46)

$${\left(A-\text{uv′}\right)}^{-1}=A^{-1}+\frac{A^{-1}uv^{\prime }A^{-1}}{1-v^{\prime }A^{-1}u}.$$ (47)

## Appendix B: Difference Between GLS Estimators

The difference between the two GLS estimators in (16) is

$$\Delta_{\beta_{j}}={\hat{\beta }}_{j,\text{in}}-{\hat{\beta }}_{j,\text{out}}=\frac{{x^{\prime }}_{j}V^{-1}y}{{x^{\prime }}_{j}V^{-1}x_{j}}-\frac{{x^{\prime }}_{j}V_{\left[-j\right]}^{-1}y}{{x^{\prime }}_{j}V_{\left[-j\right]}^{-1}x_{j}}.$$ (48)

Recalling that $V_{\left[-j\right]}^{-1}=V^{-1}+\frac{\sigma_{g}^{2}t_{j}{t^{\prime }}_{j}}{1-\sigma_{g}^{2}{x^{\prime }}_{j}t_{j}},$ where ${t^{\prime }}_{j}={x^{\prime }}_{j}V^{-1},$ and putting $s_{j}={x^{\prime }}_{j}V^{-1}x_{j}$, one can write

$$\Delta_{\beta_{j}}=\frac{{x^{\prime }}_{j}V^{-1}y}{s_{j}}-\frac{{x^{\prime }}_{j}\left(V^{-1}+\frac{\sigma_{g}^{2}t_{j}{t^{\prime }}_{j}}{1-\sigma_{g}^{2}{x^{\prime }}_{j}t_{j}}\right)y}{{x^{\prime }}_{j}\left(V^{-1}+\frac{\sigma_{g}^{2}t_{j}{t^{\prime }}_{j}}{1-\sigma_{g}^{2}{x^{\prime }}_{j}t_{j}}\right)x_{j}}=\frac{{x^{\prime }}_{j}V^{-1}y}{s_{j}}-\frac{{x^{\prime }}_{j}V^{-1}y+\frac{\sigma_{g}^{2}s_{j}{x^{\prime }}_{j}V^{-1}y}{1-\sigma_{g}^{2}s_{j}}}{s_{j}+\frac{\sigma_{g}^{2}s_{j}^{2}}{1-\sigma_{g}^{2}s_{j}}}.$$ (49)

Hence

$$\begin{array}{c} \Delta_{\beta_{j}}={x^{\prime }}_{j}V^{-1}y\left(\frac{1}{s_{j}}-\frac{1+\frac{\sigma_{g}^{2}s_{j}}{1-\sigma_{g}^{2}s_{j}}}{s_{j}+\frac{\sigma_{g}^{2}s_{j}^{2}}{1-\sigma_{g}^{2}s_{j}}}\right)={x^{\prime }}_{j}V^{-1}y\left(\frac{1}{s_{j}}-\frac{\frac{1}{1-\sigma_{g}^{2}s_{j}}}{\frac{\left(1-\sigma_{g}^{2}s_{j}\right)s_{j}+\sigma_{g}^{2}s_{j}^{2}}{1-\sigma_{g}^{2}s_{j}}}\right)\\ ={x^{\prime }}_{j}V^{-1}y\left(\frac{1}{s_{j}}-\frac{1}{s_{j}}\right)=0. \end{array}$$

The result holds provided that G=XX′; each column of X could be centered or uncentered.

## Appendix C: Difference Between BLUP Predictors

Consider BLUP after the contribution of marker *j* is removed from G, that is ${\hat{g}}_{\left[-j\right]}=\sigma_{g}^{2}G_{\left[-j\right]}V_{\left[-j\right]}^{-1}z_{j}.$ One can write, after use is made of (15) and (21)

$$\begin{array}{c} {\hat{g}}_{\left[-j\right]}=\sigma_{g}^{2}\left(G-x_{j}{x^{\prime }}_{j}\right)\left(V^{-1}+\frac{\sigma_{g}^{2}V^{-1}x_{j}{x^{\prime }}_{j}V^{-1}}{1-\sigma_{g}^{2}{x^{\prime }}_{j}V^{-1}x_{j}}\right)z_{j}\\ =\sigma_{g}^{2}GV^{-1}z_{j}+\sigma_{g}^{2}\left[\frac{\sigma_{g}^{2}GV^{-1}x_{j}{x^{\prime }}_{j}V^{-1}}{1-\sigma_{g}^{2}{x^{\prime }}_{j}V^{-1}x_{j}}-\sigma_{g}^{2}x_{j}{x^{\prime }}_{j}\left(V^{-1}+\frac{\sigma_{g}^{2}V^{-1}x_{j}{x^{\prime }}_{j}V^{-1}}{1-\sigma_{g}^{2}{x^{\prime }}_{j}V^{-1}x_{j}}\right)\right]z_{j}\\ =\sigma_{g}^{2}GV^{-1}\left(y-x_{j}{\hat{\beta }}_{j}\right)=\hat{g}. \end{array}$$ (50)

Observe that ${x^{\prime }}_{j}V^{-1}z_{j}={x^{\prime }}_{j}V^{-1}\left(y-x_{j}{\hat{\beta }}_{j}\right)=0$ because ${\hat{\beta }}_{j}=\frac{{x^{\prime }}_{j}V^{-1}y}{{x^{\prime }}_{j}V^{-1}x_{j}}.$ Thus, ${\hat{g}}_{\left[-j\right]}=\hat{g},$ and the BLUP of g is invariant with respect to removing marker *j* when constructing G.

## Appendix D: Computation of GLS with OLS

Let x be an n×1 vector containing the genotype codes for any given marker and, for simplicity, consider a model without an intercept, that is $y_{i}=x_{ij}\beta_{j}+\epsilon_{j}$ and $\epsilon_{j}∼N\left(0,\sigma_{g}^{2}+\sigma_{e}^{2}\right)$ . The GLS estimator of the marker substitution effect is

$$\hat{\beta }=\frac{x^{\prime }{\left(V^{\text{*}}\sigma_{e}^{2}\right)}^{-1}y}{x^{\prime }{\left(V^{\text{*}}\sigma_{e}^{2}\right)}^{-1}x}=\frac{x^{\prime }V^{\text{*}-1}y}{x^{\prime }V^{\text{*}-1}x},$$ (51)

and

$$Var\left(\hat{\beta }|X\right)=\frac{\sigma_{e}^{2}}{x^{\prime }V^{\text{*}-1}x},$$ (52)

with $V^{\text{*}}=G\frac{h_{g}^{2}}{1-h_{g}^{2}}+I$. Because $V^{\text{*}}$ is positive-definite, $V^{\text{*}}=C\prime C$ (Cholesky decomposition) and $V^{\text{*}-1}={\left(C\right)}^{-1}{C^{\prime }}^{-1}.$ Using this property, the data can be transformed into $z={C^{\prime }}^{-1}y,$ producing

$$z={C^{\prime }}^{-1}x\beta +{C^{\prime }}^{-1}\epsilon =k\beta +\delta ,$$ (53)

where $k={C^{\prime }}^{-1}x$ and $\delta ={C^{\prime }}^{-1}\epsilon .$ Note that $\delta ∼\left(0,I\sigma_{e}^{2}\right).$ Using this transformation, the GLS estimator can be computed as an OLS estimator applied to the transformed data z:

$$\begin{array}{c} \hat{\beta }=\frac{k\prime z}{k\prime k}\\ ={\left(x^{\prime }C^{-1}{C^{\prime }}^{-1}x\right)}^{-1}x^{\prime }C^{-1}{C^{\prime }}^{-1}y\\ ={\left(x^{\prime }V^{\text{*}-1}x\right)}^{-1}x^{\prime }V^{\text{*}-1}y, \end{array}$$ (54)

with

$$Var\left(\hat{\beta }\right)={\left(k\prime k\right)}^{-1}\sigma_{e}^{2}={\left(k^{\prime }V^{\text{*}-1}k\right)}^{-1}\sigma_{e}^{2}.$$

The GLS-derived estimator of the variance is

$${\tilde{\sigma }}_{e}^{2}=\frac{{\left(y-x\hat{\beta }\right)}^{\prime }V^{\text{*}-1}{\left(y-x\hat{\beta }\right)}^{\prime }}{n-1}=\frac{{\left(z-k\hat{\beta }\right)}^{\prime }\left(z-k\hat{\beta }\right)}{n-1},$$ (55)

which is unbiased for $\sigma_{e}^{2}.$ Our approach differed slightly because, in addition to k, we also fitted an intercept. Test statistics were as in OLS under normality, but employing the transformed phenotypes and incidence vector outlined above; significance was assessed using a Bonferroni correction with α=0.05/1279 producing a $-\log_{10}\left(p\text{value}\right)=4.408$.

## References

[bib1] Astle W. Balding D., 2009 Population structure and cryptic relatedness in genetic association studies. Stat. Sci. 24: 451–471.

[bib2] Atwell S. Huang Y. S. Vilhjalmsson B. J. Willems G. Horton M., 2010 Genome-wide association study of 107 phenotypes in *Arabidopsis thaliana* inbred lines. Nature 465: 627–631. 20336072 10.1038/nature08800 PMC3023908

[bib3] Aulchenko Y. S. de Koning D. J. Haley C., 2007 Genomewide rapid association using mixed model and regression: a fast and simple method for genomewide pedigree-based quantitative trait loci association analysis. Genetics 177: 577–585. 17660554 10.1534/genetics.107.075614 PMC2013682

[bib4] Borg I. Groenen P., 2005 *Modern Multidimensional Scaling: Theory and Applications*. Ed. 2. Springer, New York.

[bib5] Brachi B. Morris G. P. Borevitz J. O., 2011 Genome-wide association studies in plants: the missing heritability is in the field. Genome Biol. 12: 232. 22035733 10.1186/gb-2011-12-10-232 PMC3333769

[bib6] Cleveland W. S., 1979 Robust locally weighted regression and smoothing scatterplots. J. Am. Stat. Assoc. 74: 829–836.

[bib7] Crossa J. de los Campos G. Pérez P. Gianola D. Burgueño J., 2010 Prediction of genetic values of quantitative traits in plant breeding using pedigree and molecular markers. Genetics 186: 713–724. 20813882 10.1534/genetics.110.118521 PMC2954475

[bib8] de los Campos G. Sorensen D. Gianola D., 2015 Genomic heritability: what is it? PLoS Genet. 11(5): e1005048. 25942577 10.1371/journal.pgen.1005048 PMC4420472

[bib9] Falconer D. S. Mackay T. F. C., 1996 *Introduction to Quantitative Genetics*, Longman, Essex, UK.

[bib10] Gianola D., 2013 Priors in whole-genome regression: the Bayesian alphabet returns. Genetics 194: 573–596. 23636739 10.1534/genetics.113.151753 PMC3697965

[bib11] Gianola D. de los Campos G. Hill W. G. Manfredi E. Fernando R. L., 2009 Additive genetic variability and the Bayesian alphabet. Genetics 187: 347–363. 10.1534/genetics.109.103952 PMC2746159 19620397

[bib12] Gianola D. Okut H. Weigel K. A. Rosa G. J. M., 2011 Predicting complex quantitative traits with Bayesian neural networks: a case study with Jersey cows and wheat. BMC Genet. 12: 87. 21981731 10.1186/1471-2156-12-87 PMC3474182

[bib13] Gianola D. Hospital F. Verrier E., 2013 On the contribution of an additive locus to genetic variance when inheritance is multifactorial with implications on the interpretation of GWAS. Theor. Appl. Genet. 6: 1457–1472. 10.1007/s00122-013-2064-2 23508282

[bib14] Goddard M. E., 2009 Genomic selection: prediction of accuracy and maximisation of long term response. Genetica 136: 245–257. 18704696 10.1007/s10709-008-9308-0

[bib15] Gondro C. van der Werf J. Hayes B. (Editors), 2013 *Genome-Wide Association Studies and Genomic Prediction*. Springer, Berlin.

[bib16] Henderson, C. R., 1948 Estimation of general, specific and maternal combining ability in crosses among inbred lines of swine. Ph.D. Thesis, Iowa State University, Iowa.

[bib17] Henderson C. R., 1975 Best linear unbiased estimation and prediction under a selection model. Biometrics 31: 423–449. 1174616

[bib18] Henderson C. R., 1976 A simple method for computing the inverse of a numerator relationship matrix used in prediction of breeding values. Biometrics 32: 69–83.

[bib19] Henderson C. R., 1984 *Application of Linear Models in Animal Breeding*. University of Guelph, Ontario.

[bib20] Hill W. G. Weir B. S., 2011 Variation in actual relationship as a consequence of Mendelian sampling and linkage. Genet. Res. 93: 47–64. 10.1017/S0016672310000480 PMC3070763 21226974

[bib21] Janss L. de los Campos G. Sheehan N. Sorensen D., 2012 Inferences from genomic models in stratified populations. Genetics 192: 693–704. 22813891 10.1534/genetics.112.141143 PMC3454890

[bib22] Kennedy B. W. Quinton M. van Arendonk J. A. M., 1992 Estimation of effects of single genes on quantitative traits. J. Anim. Sci. 70: 2000–2012. 1644672 10.2527/1992.7072000x

[bib23] Kruskal J. B., 1964 a Multidimensional scaling by optomizing goodness of fit to nonmetric hypotheses. Psychometrika 29: 1–28.

[bib24] Kruskal J. B., 1964 b Nometric multidimensional scaling: a numerical method. Psychometrika 29: 115–129.

[bib25] Legarra A., 2015 Comparing estimates of genetic variance across different relationship models. Theor. Popul. Biol. 107: 26–30. 26341159 10.1016/j.tpb.2015.08.005

[bib26] Lipka A. E. Kandianis C. B. Hudson M. E. Yu J. Drnevich J., 2015 From association to prediction: statistical methods for the dissection and selection of complex traits in plants. Curr. Opin. Plant Biol. 24: 110–118. 25795170 10.1016/j.pbi.2015.02.010

[bib27] Lo A. Chernoff H. Zheng T. Lo S-H., 2015 Why significant variables aren’t automatically good predictors. Proc. Natl. Acad. Sci. USA 112: 13892–13897. 26504198 10.1073/pnas.1518285112 PMC4653162

[bib28] Long N. Gianola D. Rosa G. J. M. Weigel K. A., 2011 Application of support vector regression to genome-assisted prediction of quantitative traits. Theor. Appl. Genet. 123: 1065–1074. 21739137 10.1007/s00122-011-1648-y

[bib29] Lynch M. Walsh B., 1998 *Genetics and Analysis of Quantitative Traits*, Sinauer Associates, Inc., Sunderland, MA.

[bib30] Maher B., 2008 Personal genomes: the case of the missing heritability. Nature 456: 18–21. 18987709 10.1038/456018a

[bib31] Manolio T. A. Collins F. S. Cox N. J. Goldstein D. B. Hindorff L. A., 2009 Finding the missing heritability of complex diseases. Nature 461: 747–753. 19812666 10.1038/nature08494 PMC2831613

[bib32] Meyer K. Tier B., 2012 “SNP Snappy”: a strategy for fast genome-wide association studies fitting a full mixed model. Genetics 190: 275–277. 22021386 10.1534/genetics.111.134841 PMC3249377

[bib33] Neimann-Sorensen A. Robertson A., 1961 The association between blood groups and several production characteristics in three Danish cattle breeds. Acta Agriculturae Scandinavica 11: 163–196.

[bib34] Nejati-Javaremi A. Smith C. Gibson J. P., 1997 Effect of total allelic relationship on accuracy of evaluation and response to selection. J. Anim. Sci. 7: 1738–1745. 10.2527/1997.7571738x 9222829

[bib35] Norborg M. Hu T. T. Ishino Y. Javeri J. Toomajian C., 2005 The pattern of polymorphism in *Arabidopsis thaliana*. PLoS Biol. 3(7): e196. 15907155 10.1371/journal.pbio.0030196 PMC1135296

[bib36] Pérez P. de los Campos G., 2014 Genome-wide regression and prediction with the BGLR statistical package. Genetics 198: 483–495. 25009151 10.1534/genetics.114.164442 PMC4196607

[bib37] Price A. L. Zaitlen N. A. Reich D. Patterson N., 2010 New approaches to population stratification in genome-wide association studies. Nat. Rev. Genet. 11: 459–463. 20548291 10.1038/nrg2813 PMC2975875

[bib38] Rincent R. Moreau L. Monod H. Kuhn E. Melchinger A. E., 2014 Recovering power in association mapping panels with variable levels of linkage disequilibrium. Genetics 197: 375–387. 24532779 10.1534/genetics.113.159731 PMC4012494

[bib39] Searle S. R., 1974 Prediction, mixed models and variance components, pp. 229–266 in Reliability and Biometry, edited by Proschan F. Serfling R. I. Society for Industrial and Applied Mathematics (SIAM), Philadelphia.

[bib40] Seber G. A. F. Lee A. J., 2003 *Linear Regression Analysis*, Wiley-Blackwell, New York.

[bib41] Shmueli G., 2010 To explain or to predict? Stat. Sci. 25: 289–310.

[bib42] Stahl E. A. Wegmann D. Trynka G. Gutierrez-Achury J. Do R., 2012 Bayesian inference analyses of the polygenic architecture of rheumatoid arthritis. Nat. Genet. 44: 483–489. 22446960 10.1038/ng.2232 PMC6560362

[bib43] Sun G. Zhu C. Kramer M. H. Yang S. S. Song W., 2010 Variation explained in mixed-model association mapping. Heredity 105: 333–340. 20145669 10.1038/hdy.2010.11

[bib44] Teyssèdre S. Elsen J. M. Ricard A., 2012 Statistical distributions of test statistics used for quantitative trait association mapping in structured populations. Genet. Sel. Evol. 44: 32. 23146127 10.1186/1297-9686-44-32 PMC3817592

[bib45] Van Raden P. M., 2008 Efficient methods to compute genomic predictions. J. Dairy Sci. 91: 4414–4423. 18946147 10.3168/jds.2007-0980

[bib46] Wimmer V. Albrecht T. Auinger H. J. Schön C. C., 2012 Synbreed: a framework for the analysis of genomic prediction data using R. Bioinformatics 28: 2086–2087. 22689388 10.1093/bioinformatics/bts335

[bib47] Wimmer V. Lehermeier C. Albrecht T. Auinger H. J. Wang Y., 2013 Genome-wide prediction of traits with different genetic architecture through efficient variable selection. Genetics 195: 573–587. 23934883 10.1534/genetics.113.150078 PMC3781982

[bib48] Yang J. Benyamin B. McEvoy B. P. Gordon S. Henders A. K., 2010 Common SNPs explain a large proportion of the heritability for human height. Nat. Genet. 42: 565–569. 20562875 10.1038/ng.608 PMC3232052

[bib49] Yang J. Manolio T. A. Pasquale L. R. Boerwinkle E. Caporaso N., 2011 Genome partitioning of genetic variation for complex traits using common SNPs. Nat. Genet. 43: 519–525. 10.1038/ng.823 PMC4295936 21552263

[bib50] Yu J. Pressoir G. Briggs W. H. Vroh Bi I. Yamasaki M., 2006 A unified mixed model for association mapping that accounts for multiple levels of relatedness. Nat. Genet. 38: 203–208. 16380716 10.1038/ng1702

[bib51] Zhu C. Yu J., 2009 Nonmetric multidimensional scaling corrects for population structure in association mapping with different sample types. Genetics 182: 875–888. 19414565 10.1534/genetics.108.098863 PMC2710166

