# Supplementary material for: Genome-Wide Association Studies with a Genomic Relationship Matrix: A Case Study with Wheat and Arabidopsis
Source: G3 (Bethesda). 2016 Aug 11;6(10):3241–56. doi: 10.1534/g3.116.034256 (PMC5068945; doi:10.1534/g3.116.034256)
Supplement: Supplemental Material [file supp_6_10_3241__index.html]

Genome-Wide Association Studies with a Genomic Relationship Matrix: A Case Study with Wheat and Arabidopsis — Supplemental Material 

# Genome-Wide Association Studies with a Genomic Relationship Matrix: A Case Study with Wheat and *Arabidopsis*

## Supplemental Material for Gianola *et al.*, 2016

**Files in this Data Supplement:**

- File S1 - Toy examples. (85 KB)
