# Supplementary material for: Genome-Wide Association Studies with a Genomic Relationship Matrix: A Case Study with Wheat and Arabidopsis
Source: G3 (Bethesda). 2016 Aug 11;6(10):3241–56. doi: 10.1534/g3.116.034256 (PMC5068945; doi:10.1534/g3.116.034256)
Supplement: Supplemental Material [file supp_g3.116.034256_FileS1.pdf]

1 Supplementary File: Toy examples

1.1 Example 1: forming inverses of genomic relationship matrices

There are 3 individuals with phenotypes, each genotyped with 4 markers coded as $-1, 0, 1$ for aa, Aa and AA genotypes, at the first locus, respectively, and so on. Markers are not standardized and the information is

$$\mathbf{y} = \begin{bmatrix} 1 \\ 3 \\ 4 \end{bmatrix}, \ \mathbf{X} = \begin{bmatrix} -1 & 0 & 0 & -1 \\ -1 & 1 & 0 & 1 \\ 1 & 1 & 1 & -1 \end{bmatrix}. \tag{1}$$

Here $\mathbf{G} = \mathbf{XX}'$ is 3×3 with

$$\mathbf{G} = \mathbf{XX}' = \begin{bmatrix} 2 & 0 & 0 \\ 0 & 3 & -1 \\ 0 & -1 & 4 \end{bmatrix}, \ \mathbf{G}^{-1} = \begin{bmatrix} \frac{1}{2} & 0 & 0 \\ 0 & \frac{4}{11} & \frac{1}{11} \\ 0 & \frac{1}{11} & \frac{3}{11} \end{bmatrix}. \tag{2}$$

Individuals 2 and 3 are "genomically related" (the off-diagonal is -1) and $rank\left(\mathbf{G}\right) = 3$. Using developments in the body of the paper, removal of the first marker from $\mathbf{X}$ produces

$$\mathbf{G}_{[-1]} = \begin{bmatrix} 1 & -1 & 1 \\ -1 & 2 & 0 \\ 1 & 0 & 3 \end{bmatrix}, \ \mathbf{G}_{[-1]}^{-1} = \begin{bmatrix} 6 & 3 & -2 \\ 3 & 2 & -1 \\ -2 & -1 & 1 \end{bmatrix}. \tag{3}$$

Next, compute the inverse as described in the main body so that

$$\mathbf{G}_{[-1]}^{-1} = \mathbf{G}^{-1}\left[\mathbf{I} + \frac{\mathbf{1}}{1 - \mathbf{x}_1'\mathbf{t}_1}\mathbf{x}_1\mathbf{t}_1'\right]. \tag{4}$$

Here,

$$\begin{aligned}
\mathbf{t}_1 &= \mathbf{G}^{-1}\mathbf{x}_1 = \begin{bmatrix} \frac{1}{2} & 0 & 0 \\ 0 & \frac{4}{11} & \frac{1}{11} \\ 0 & \frac{1}{11} & \frac{3}{11} \end{bmatrix} \begin{bmatrix} -1 \\ -1 \\ 1 \end{bmatrix} = \begin{bmatrix} -\frac{1}{2} \\ -\frac{3}{11} \\ \frac{2}{11} \end{bmatrix}, \\
\mathbf{x}_1\mathbf{t}_1' &= \begin{bmatrix} -1 \\ -1 \\ 1 \end{bmatrix} \begin{bmatrix} -\frac{1}{2} \\ -\frac{3}{11} \\ \frac{2}{11} \end{bmatrix}' = \begin{bmatrix} \frac{1}{2} & \frac{3}{11} & -\frac{2}{11} \\ \frac{1}{2} & \frac{3}{11} & -\frac{2}{11} \\ -\frac{1}{2} & -\frac{3}{11} & \frac{2}{11} \end{bmatrix}, \\
1 - \mathbf{x}_1'\mathbf{t}_1 &= 1 - \begin{bmatrix} -1 \\ -1 \\ 1 \end{bmatrix}' \begin{bmatrix} -\frac{1}{2} \\ -\frac{3}{11} \\ \frac{2}{11} \end{bmatrix} = \frac{1}{22}, \\
\frac{1}{1 - \mathbf{x}_1'\mathbf{t}_1}\mathbf{x}_1\mathbf{t}_1' &= 22 \begin{bmatrix} \frac{1}{2} & \frac{3}{11} & -\frac{2}{11} \\ \frac{1}{2} & \frac{3}{11} & -\frac{2}{11} \\ -\frac{1}{2} & -\frac{3}{11} & \frac{2}{11} \end{bmatrix} = \begin{bmatrix} 11 & 6 & -4 \\ 11 & 6 & -4 \\ -11 & -6 & 4 \end{bmatrix}.
\end{aligned}$$

Thus

$$\mathbf{G}_{[-1]}^{-1} = \begin{bmatrix} \frac{1}{2} & 0 & 0 \\ 0 & \frac{4}{11} & \frac{1}{11} \\ 0 & \frac{1}{11} & \frac{3}{11} \end{bmatrix} \begin{bmatrix} 1+11 & 6 & -4 \\ 11 & 1+6 & -4 \\ -11 & -6 & 1+4 \end{bmatrix} = \begin{bmatrix} 6 & 3 & -2 \\ 3 & 2 & -1 \\ -2 & -1 & 1 \end{bmatrix}. \quad (5)$$

The result is the same as that obtained by direct inversion of $\mathbf{G}_{[-1]}$, as given in (3).

Next, remove the second marker instead of the first from $\mathbf{X}$, obtaining

$$\mathbf{G}_{[-2]} = \begin{bmatrix} 2 & 0 & 0 \\ 0 & 2 & -2 \\ 0 & -2 & 3 \end{bmatrix}, \ \mathbf{G}_{[-2]}^{-1}, = \begin{bmatrix} \frac{1}{2} & 0 & 0 \\ 0 & \frac{3}{2} & 1 \\ 0 & 1 & 1 \end{bmatrix}. \quad (6)$$

Form the inverse as

$$\mathbf{G}_{[-2]}^{-1} = \mathbf{G}^{-1}\left[\mathbf{I} + \frac{\mathbf{1}}{1-\mathbf{x}_2'\mathbf{t}_2}\mathbf{x}_2\mathbf{t}_2'\right],$$

with

$$\mathbf{t}_2 = \begin{bmatrix} 0 \\ \frac{5}{11} \\ \frac{4}{11} \end{bmatrix};\ \mathbf{x}_2'\mathbf{t}_2 = \frac{9}{11};\ \mathbf{x}_2\mathbf{t}_2' = \begin{bmatrix} 0 & 0 & 0 \\ 0 & \frac{5}{11} & \frac{4}{11} \\ 0 & \frac{5}{11} & \frac{4}{11} \end{bmatrix},$$

giving

$$\mathbf{G}_{[-2]}^{-1} = \begin{bmatrix} \frac{1}{2} & 0 & 0 \\ 0 & \frac{4}{11} & \frac{1}{11} \\ 0 & \frac{1}{11} & \frac{3}{11} \end{bmatrix} \begin{bmatrix} 1 & 0 & 0 \\ 0 & 1+\frac{5}{2} & 2 \\ 0 & \frac{5}{2} & 1+2 \end{bmatrix} = \begin{bmatrix} \frac{1}{2} & 0 & 0 \\ 0 & \frac{3}{2} & 1 \\ 0 & 1 & 1 \end{bmatrix}, \qquad (7)$$

as obtained in (6) with direct inversion.

1.2 Example 2: Regressions on markers via OLS

The setting is that of Example 1; let $\sigma_e^2 = \sigma_g^2 = 1$ so that genomic heritability is $\frac{1}{2}$. First, we do single marker regression for the first two markers using OLS. The esimates are

$$\beta_1^{SMR} = \frac{\mathbf{x}_1'\mathbf{y}}{\mathbf{x}_1'\mathbf{x}_1} = 0;\ Var\left(\beta_1^{SMR}\right) = \frac{1}{\mathbf{x}_1'\mathbf{x}_1} = \frac{1}{3}, \qquad (8)$$

and

$$\beta_2^{SMR} = \frac{\mathbf{x}_2'\mathbf{y}}{\mathbf{x}_2'\mathbf{x}_2} = \frac{7}{2};\ Var\left(\beta_2^{SMR}\right) = \frac{1}{\mathbf{x}_2'\mathbf{x}_2} = \frac{1}{2}. \qquad (9)$$

The OLS multiple regression (MR) on these two markers fitted together is

$$\begin{aligned}\begin{bmatrix}\beta_1\\ \beta_2\end{bmatrix}^{MR} &= \left(\begin{bmatrix}\mathbf{x}_1 & \mathbf{x}_2\end{bmatrix}'\begin{bmatrix}\mathbf{x}_1 & \mathbf{x}_2\end{bmatrix}\right)^{-1}\left(\begin{bmatrix}\mathbf{x}_1 & \mathbf{x}_2\end{bmatrix}'\mathbf{y}\right)^{-1}\\ &= \begin{bmatrix}3 & 0\\ 0 & 2\end{bmatrix}^{-1}\begin{bmatrix}0\\ 7\end{bmatrix} = \begin{bmatrix}0\\ \frac{7}{2}\end{bmatrix}, \qquad (10)\end{aligned}$$

giving the same estimates as in SMR because $\mathbf{x}_1$ and $\mathbf{x}_2$ are orthogonal. Hence, as before,

$$Var\begin{bmatrix}\beta_1\\ \beta_2\end{bmatrix}^{SMR} = \begin{bmatrix}3 & 0\\ 0 & 2\end{bmatrix}^{-1} = \begin{bmatrix}\frac{1}{3} & 0\\ 0 & \frac{1}{2}\end{bmatrix}. \qquad (11)$$

With real data, however, markers are seldom mutually orthogonal because linkage disequilibrium creates co-linearity among columns of $\mathbf{X}$.

1.3 Example 3: GLS with a genomic relationship matrix

Because of molecular similarity between marker genotypes in pairs of individuals, observations are correlated to a degree conveyed by the genomic relationship matrix. If the first marker is removed to be tested as a fixed effect, the phenotypic variance-covariance matrix and its inverse are

$$\begin{aligned}\mathbf{V}_{[-1]} &= \mathbf{G}_{[-1]}\sigma_g^2 + \mathbf{I}\sigma_e^2 = \begin{bmatrix}1 & -1 & 1\\ -1 & 2 & 0\\ 1 & 0 & 3\end{bmatrix} + \begin{bmatrix}1 & 0 & 0\\ 0 & 1 & 0\\ 0 & 0 & 1\end{bmatrix} = \begin{bmatrix}2 & -1 & 1\\ -1 & 3 & 0\\ 1 & 0 & 4\end{bmatrix};\\ \mathbf{V}_{[-1]}^{-1} &= \begin{bmatrix}\frac{12}{17} & \frac{4}{17} & -\frac{3}{17}\\ \frac{4}{17} & \frac{7}{17} & -\frac{1}{17}\\ -\frac{3}{17} & -\frac{1}{17} & \frac{5}{17}\end{bmatrix}. \qquad (12)\end{aligned}$$

For the second marker

$$\mathbf{V}_{[-2]} = \mathbf{G}_{[-2]}\sigma_g^2 + \mathbf{I}\sigma_e^2 = \begin{bmatrix} 2 & 0 & 0 \\ 0 & 2 & -2 \\ 0 & -2 & 3 \end{bmatrix} + \begin{bmatrix} 1 & 0 & 0 \\ 0 & 1 & 0 \\ 0 & 0 & 1 \end{bmatrix} = \begin{bmatrix} 3 & 0 & 0 \\ 0 & 3 & -2 \\ 0 & -2 & 4 \end{bmatrix};$$

$$\mathbf{V}_{[-2]}^{-1} = \begin{bmatrix} \frac{1}{3} & 0 & 0 \\ 0 & \frac{1}{2} & \frac{1}{4} \\ 0 & \frac{1}{4} & \frac{3}{8} \end{bmatrix}. \qquad (13)$$

Note that removal of the first marker creates a negative covariance between observations in individuals 1 and 2, while absence of marker 2 yields a null one.

The GLS-SMR estimates for markers 1 and 2, using results developed in the main body, are

$$\widehat{\beta}_{[-1]} = \frac{\mathbf{x}_1'\mathbf{V}_{[-1]}^{-1}\mathbf{y}}{\mathbf{x}_1'\mathbf{V}_{[-1]}^{-1}\mathbf{x}_1} = \frac{\begin{bmatrix} -1 \\ -1 \\ 1 \end{bmatrix}' \begin{bmatrix} \frac{12}{17} & \frac{4}{17} & -\frac{3}{17} \\ \frac{4}{17} & \frac{7}{17} & -\frac{1}{17} \\ -\frac{3}{17} & -\frac{1}{17} & \frac{5}{17} \end{bmatrix} \begin{bmatrix} 1 \\ 3 \\ 4 \end{bmatrix}}{\begin{bmatrix} -1 \\ -1 \\ 1 \end{bmatrix}' \begin{bmatrix} \frac{12}{17} & \frac{4}{17} & -\frac{3}{17} \\ \frac{4}{17} & \frac{7}{17} & -\frac{1}{17} \\ -\frac{3}{17} & -\frac{1}{17} & \frac{5}{17} \end{bmatrix} \begin{bmatrix} -1 \\ -1 \\ 1 \end{bmatrix}} = -\frac{19}{40}; \qquad (14)$$

$$Var\left(\widehat{\beta}_{[-1]}\right) = \frac{17}{40} = 0.425, \qquad (15)$$

and

$$\widehat{\beta}_{[-2]} = \frac{\mathbf{x}_2'\mathbf{V}_{[-2]}^{-1}\mathbf{y}}{\mathbf{x}_2'\mathbf{V}_{[-2]}^{-1}\mathbf{x}_2} = \frac{\begin{bmatrix}0\\1\\1\end{bmatrix}'\begin{bmatrix}\frac{1}{3}&0&0\\0&\frac{1}{2}&\frac{1}{4}\\0&\frac{1}{4}&\frac{3}{8}\end{bmatrix}\begin{bmatrix}1\\3\\4\end{bmatrix}}{\begin{bmatrix}0\\1\\1\end{bmatrix}'\begin{bmatrix}\frac{1}{3}&0&0\\0&\frac{1}{2}&\frac{1}{4}\\0&\frac{1}{4}&\frac{3}{8}\end{bmatrix}\begin{bmatrix}0\\1\\1\end{bmatrix}} = \frac{38}{11}; \quad (16)$$

$$Var\left(\widehat{\beta}_{[-2]}\right) = \frac{8}{11}. \quad (17)$$

Note that the GLS estimate of the regression on marker 1 is not 0, contrary to what is given by OLS in (8), and that the variances of the estimates are understated by OLS. For marker 1, the variance of the GLS estimate, $\frac{17}{40}$, is about 30% larger than the $\frac{1}{3}$ indicated by OLS. For marker 2, OLS gives $\frac{1}{2}$ as variance, which is $\frac{1}{2}/\frac{8}{11} = \frac{11}{16}$ of the GLS variance, an understatement of about 31%. The stylized example illustrates that ignoring correlations between observations may produce different point and interval estimates of marker effects.

Consider now fitting the two markers together while maintaining their contribution to the genomic relationship matrix, so that $\mathbf{G}$ is as in (2) and the variance-covariance matrix of the observations is taken to be $\mathbf{V} = \mathbf{G}\sigma_e^2 + \mathbf{I}\sigma_g^2$.

Recalling that $\sigma_g^2 = \sigma_e^2 = 1$ the resulting GLS estimate is

$$\begin{aligned}
&\begin{bmatrix} \beta_1 \\ \beta_2 \end{bmatrix}^{GLS} \\
&= \left(\begin{bmatrix} \mathbf{x}_1 & \mathbf{x}_2 \end{bmatrix}' \mathbf{V}^{-1} \begin{bmatrix} \mathbf{x}_1 & \mathbf{x}_2 \end{bmatrix} \right)^{-1} \left(\begin{bmatrix} \mathbf{x}_1 & \mathbf{x}_2 \end{bmatrix}' \mathbf{V}^{-1} \mathbf{y} \right) \\
&= \left(\begin{bmatrix} -1 & 0 \\ -1 & 1 \\ 1 & 1 \end{bmatrix}' \begin{bmatrix} 2+1 & 0 & 0 \\ 0 & 3+1 & -1 \\ 0 & -1 & 4+1 \end{bmatrix}^{-1} \begin{bmatrix} -1 & 0 \\ -1 & 1 \\ 1 & 1 \end{bmatrix} \right)^{-1} \times \\
&\quad \left(\begin{bmatrix} -1 & 0 \\ -1 & 1 \\ 1 & 1 \end{bmatrix}' \begin{bmatrix} 2+1 & 0 & 0 \\ 0 & 3+1 & -1 \\ 0 & -1 & 4+1 \end{bmatrix}^{-1} \begin{bmatrix} 1 \\ 3 \\ 4 \end{bmatrix} \right) \\
&= \begin{bmatrix} \frac{33}{23} & \frac{3}{23} \\ \frac{3}{23} & \frac{40}{23} \end{bmatrix} \begin{bmatrix} -\frac{1}{3} \\ 2 \end{bmatrix} = \begin{bmatrix} -\frac{5}{23} \\ \frac{79}{23} \end{bmatrix}, \qquad (18)
\end{aligned}$$

and

$$Var\left(\begin{bmatrix} \beta_1 \\ \beta_2 \end{bmatrix}^{GLS} \right) = \begin{bmatrix} \frac{33}{23} & \frac{3}{23} \\ \frac{3}{23} & \frac{40}{23} \end{bmatrix} \approx \begin{bmatrix} 1.4348 & 0.1304 \\ 0.1304 & 1.7391 \end{bmatrix}. \qquad (19)$$

These estimates and their variances are different from the single marker OLS and the "marker-out" GLS estimates; for example, the single marker GLS estimate for markers 1 and 2 are $-\frac{19}{40} = -0.475$ and $\frac{38}{11} \approx 3.45$, respectively, while estimates found with the two markers fitted together are $\left(-\frac{5}{23} \approx -0.22, \frac{79}{23} \approx 3.43\right)$.

We could amend $\mathbf{G}$ by forming another genomic relationship matrix that excludes both markers 1 and 2:

$$\mathbf{G}_{[-1-2]} = \mathbf{X}_{[-1-2]}\mathbf{X}'_{[-1-2]} = \begin{bmatrix} 0 & -1 \\ 0 & 1 \\ 1 & -1 \end{bmatrix} \begin{bmatrix} 0 & -1 \\ 0 & 1 \\ 1 & -1 \end{bmatrix}' = \begin{bmatrix} 1 & -1 & 1 \\ -1 & 1 & -1 \\ 1 & -1 & 2 \end{bmatrix}.$$

However, $rank\left(\mathbf{G}_{[-1-2]}\right) = 2$ so its inverse is not unique. However

$$\mathbf{V}_{[-1-2]}^{-1} = \left(\begin{bmatrix} 1 & -1 & 1 \\ -1 & 1 & -1 \\ 1 & -1 & 2 \end{bmatrix} + \begin{bmatrix} 1 & 0 & 0 \\ 0 & 1 & 0 \\ 0 & 0 & 1 \end{bmatrix} \right)^{-1} = \begin{bmatrix} \frac{5}{7} & \frac{2}{7} & -\frac{1}{7} \\ \frac{2}{7} & \frac{5}{7} & \frac{1}{7} \\ -\frac{1}{7} & \frac{1}{7} & \frac{3}{7} \end{bmatrix},$$

so the GLS regression on marker 3 is estimable.

1.4 Example 4: Finding the GLS estimates with the mixed model equations

First, we fit the first two markers together and verify that results are as in (18) and (19); all markers are used for forming $\mathbf{G}$. The mixed model equations, with markers 1 and 2 fitted simultaneously as fixed effects and both variance components equal to 1, are

$$\begin{bmatrix} 3 & 0 & -1 & -1 & 1 \\ 0 & 2 & 0 & 1 & 1 \\ -1 & 0 & 1+\frac{1}{2} & 0 & 0 \\ -1 & 1 & 0 & 1+\frac{4}{11} & \frac{1}{11} \\ 1 & 1 & 0 & \frac{1}{11} & 1+\frac{3}{11} \end{bmatrix} \begin{bmatrix} \widetilde{\beta}_1 \\ \widetilde{\beta}_2 \\ \widetilde{g}_1 \\ \widetilde{g}_2 \\ \widetilde{g}_3 \end{bmatrix} = \begin{bmatrix} 0 \\ 7 \\ 1 \\ 3 \\ 4 \end{bmatrix}. \tag{20}$$

Here, $\widetilde{\beta_1}$ and $\widetilde{\beta_2}$ are the GLS estimates of the corresponding regression coefficients, and $\widetilde{g}_i$ $(i = 1, 2, 3)$ is the GBLUP of g_i. The solutions are

$$\begin{bmatrix} \widetilde{\beta}_1 \\ \widetilde{\beta}_2 \\ \widetilde{g}_1 \\ \widetilde{g}_2 \\ \widetilde{g}_3 \end{bmatrix} = \begin{bmatrix} -\frac{5}{23} \\ \frac{79}{23} \\ \frac{12}{23} \\ -\frac{12}{23} \\ \frac{15}{23} \end{bmatrix} \approx \begin{bmatrix} -0.2174 \\ 3.4348 \\ 0.5217 \\ -0.5217 \\ 0.6522 \end{bmatrix}; \tag{21}$$

the GLS estimates are as in (18), as it should.

Next we illustrate how the single marker GLS estimates are obtained. Removing marker 1 when forming the genomic relationship matrix produces the system

$$\begin{bmatrix} \widetilde{\beta}_{[-1]} \\ \widetilde{g}_{1,[-1]} \\ \widetilde{g}_{2,[-1]} \\ \widetilde{g}_{3,[-1]} \end{bmatrix} = \begin{bmatrix} 3 & -1 & -1 & 1 \\ -1 & 1+6 & 3 & -2 \\ -1 & 3 & 1+2 & -1 \\ 1 & -2 & -1 & 1+1 \end{bmatrix}^{-1} \begin{bmatrix} 0 \\ 1 \\ 3 \\ 4 \end{bmatrix} = \begin{bmatrix} -\frac{19}{40} \\ \frac{7}{20} \\ \frac{13}{8} \\ \frac{17}{5} \end{bmatrix} = \begin{bmatrix} -0.475 \\ 0.35 \\ 1.625 \\ 3.4 \end{bmatrix}, \tag{22}$$

with

$$\begin{bmatrix} 3 & -1 & -1 & 1 \\ -1 & 1+6 & 3 & -2 \\ -1 & 3 & 1+2 & -1 \\ 1 & -2 & -1 & 1+1 \end{bmatrix}^{-1} = \begin{bmatrix} \frac{17}{40} & -\frac{1}{20} & \frac{1}{8} & -\frac{1}{5} \\ -\frac{1}{20} & \frac{3}{10} & -\frac{1}{4} & \frac{1}{5} \\ \frac{1}{8} & -\frac{1}{4} & \frac{5}{8} & 0 \\ -\frac{1}{5} & \frac{1}{5} & 0 & \frac{4}{5} \end{bmatrix}.$$

This verifies that the GLS estimate (-0.475) and its variance $\left(\frac{17}{40}\right)$ are as obtained in Example 3. Removing the second marker when forming the relationship matrix gives

$$\begin{bmatrix} \widetilde{\beta}_{[-2]} \\ \widetilde{g}_{1,[-2]} \\ \widetilde{g}_{2,[-2]} \\ \widetilde{g}_{3,[-2]} \end{bmatrix} = \begin{bmatrix} 2 & 0 & 1 & 1 \\ 0 & 1+\frac{1}{2} & 0 & 0 \\ 1 & 0 & 1+\frac{3}{2} & 1 \\ 1 & 0 & 1 & 1+1 \end{bmatrix}^{-1} \begin{bmatrix} 7 \\ 1 \\ 3 \\ 4 \end{bmatrix} = \begin{bmatrix} \frac{38}{11} \\ \frac{2}{3} \\ -\frac{4}{11} \\ \frac{5}{11} \end{bmatrix}, \tag{23}$$

with

$$\begin{bmatrix} 2 & 0 & 1 & 1 \\ 0 & 1+\frac{1}{2} & 0 & 0 \\ 1 & 0 & 1+\frac{3}{2} & 1 \\ 1 & 0 & 1 & 1+1 \end{bmatrix}^{-1} = \begin{bmatrix} \frac{8}{11} & 0 & -\frac{2}{11} & -\frac{3}{11} \\ 0 & \frac{2}{3} & 0 & 0 \\ -\frac{2}{11} & 0 & \frac{6}{11} & -\frac{2}{11} \\ -\frac{3}{11} & 0 & -\frac{2}{11} & \frac{8}{11} \end{bmatrix},$$

so that $\widetilde{\beta}_{[-2]} = \frac{38}{11}$ and $Var\left(\widetilde{\beta}_{[-2]}\right) = \frac{8}{11}$ are as found earlier in (16) and (17), respectively.
